# RGG-box in hnRNPA1 specifically recognizes the telomere G-quadruplex DNA and enhances the G-quadruplex unfolding ability of UP1 domain

**DOI:** 10.1093/nar/gky854

**Published:** 2018-09-22

**Authors:** Meenakshi Ghosh, Mahavir Singh

**Affiliations:** 1 Molecular Biophysics Unit, Indian Institute of Science, Bengaluru 560012, India; 2 NMR Research Centre, Indian Institute of Science, Bengaluru 560012, India

## Abstract

hnRNPA1 is a member of heteronuclear ribonucleoproteins that has been shown to promote telomere elongation apart from its roles in RNA transport and alternative splicing. It is a modular protein with an N-terminal domain called UP1 that consists of two RNA Recognition Motifs (RRM1 and RRM2 domains) and a C-terminal region that harbors functional motifs such as RGG-box, a prion-like domain, and a nuclear shuttling sequence. UP1 has been reported to bind and destabilize telomeric DNA G-quadruplexes and thereby participate in DNA telomere remodeling. An RGG-box motif that consists of four RGG repeats (containing arginine and glycine residues) is located C-terminal to the UP1 domain and constitutes an additional nucleic acid and protein-binding domain. However, the precise role of the RGG-box of hnRNPA1 in telomere DNA recognition and G-quadruplex DNA unfolding remains unexplored. Here, we show that the isolated RGG-box interacts specifically with the structured telomere G-quadruplex DNA but not with the single-stranded DNA. Further the interaction of the RGG-box with the G-quadruplex DNA is dependent on the loop nucleotides of the G-quadruplex. Finally, we show that the RGG-box enhances the G-quadruplex unfolding activity of the adjacent UP1 domain. We propose that UP1 and RGG-box act synergistically to achieve complete telomere G-quadruplex DNA unfolding.

## INTRODUCTION

Linear ends of the eukaryotic chromosomes have specialized nucleoprotein complex consisting of telomere DNA and telomere DNA binding proteins. Telomere nucleoprotein structures prevent chromosome ends from degradation and end-to-end fusion with neighboring chromosomes (1). The length of the human telomeric DNA is ∼9–15 kb that terminates in 3′ G-rich overhang of ∼50–300 nucleotides. The 3′ G-rich overhang forms a lasso like t-loop, in which the 3′ overhang loops back and invades the double stranded region by displacing the original strand thereby forming a D-loop (the displacement loop) structure. Further the double and single stranded regions of telomere DNA are bound by a specific set of proteins collectively called the shelterin complex proteins (2). A specialized reverse transcriptase called the telomerase holoenzyme uses its RNA subunit as a template to extend the telomere DNA repeats (1,3). The single stranded 3′ G-overhang telomere DNA repeats are known to form higher order G-quadruplex structures both *in vitro* and *in vivo*. These higher order DNA structures have been found to inhibit the telomerase activity. Therefore, G-quadruplex binding and stabilizing molecules are considered interesting targets for anticancer drugs (4). Together, the higher order t/D loop, the shelterin complex, and the G-quadruplexes provide stability to the telomere ends (2).

The telomere DNA remodeling during telomere DNA replication by DNA polymerase and elongation by the telomerase requires dismantling and unfolding of the higher order t/D-loop and G-quadruplex structures (3,5). Indeed a number of proteins are known to help in t/D loop disassembly and G-quadruplex unfolding. While many of these proteins have ATP dependent helicase activities (such as WRN, BLM and RTEL1 helicases), there are other proteins such as hnRNPA1, hPOT1 and RPA1 that destabilize DNA G-quadruplexes in a non-enzymatic fashion (3,5–8).

**Figure 1.**
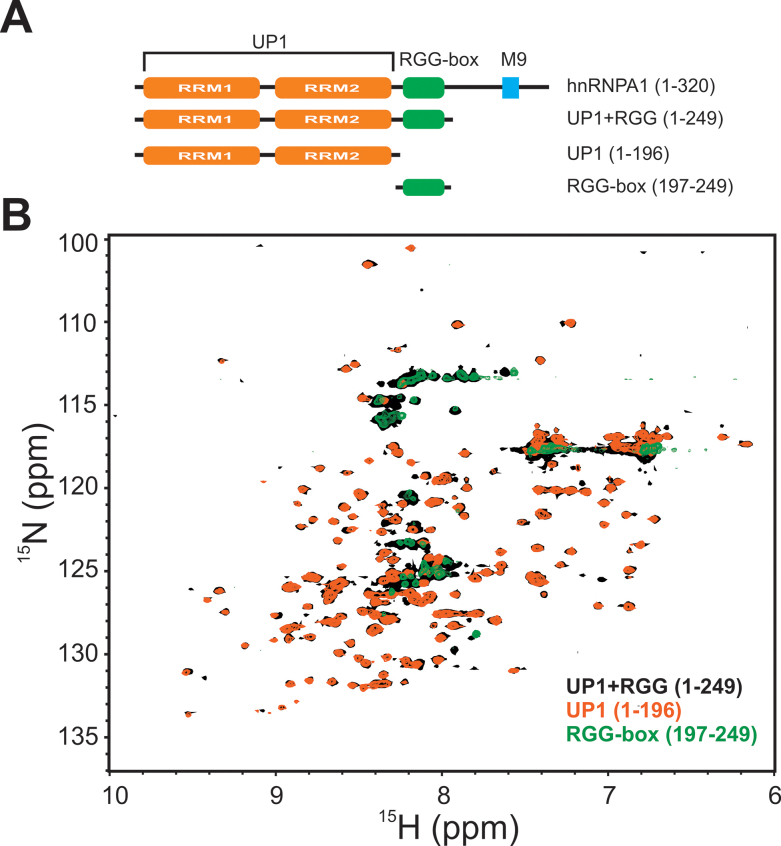
Different constructs of hnRNPA1 domains used in this study. (**A**) Domain architecture of hnRNPA1. Different constructs used in this study are shown below the full-length protein along with the domain boundaries. (**B**) Overlay of the 2D ^15^N–^1^H HSQC spectra of UP1 (orange), UP1+RGG (black) and RGG-box (green).

Heterogenous nuclear ribonucleoprotein A1 (hnRNPA1) is an abundant nuclear protein involved in various aspects of RNA metabolism in the cell that include alternative splicing, mRNA transport, trafficking, and export (9–11). Besides these roles, hnRNPA1 has specific roles at the telomere ends. Deficiency of hnRNPA1 in cell lines has been directly associated with the shortening of telomere DNA, which is restored upon over expression of hnRNPA1 (12,13). The most abundant isoform of hnRNPA1 is a protein containing 320 residues that consists of an N-terminal domain called UP1, comprising of two RNA recognition motifs (RRM1 and RRM2 domains) and a C-terminal domain (CTD). The CTD contains an RGG-box consisting of four RGG repeats, a prion-like domain, and a nuclear shuttling sequence called the M9 sequence (Figure 1A) (10). The prion-like domain has been shown to promote protein-protein interaction leading to stress granule assembly and pathological protein fibrilization (14–16). While the N-terminal UP1 domain is studied in detail for both structure and function, the functional details of the CTD region has just begun to emerge (17).

Three distinct activities have been associated with the UP1 domain: Firstly, it interacts with the single-stranded DNA (ssDNA) and G-quadruplex DNA structures; secondly, it interacts with the single-stranded RNA (ssRNA) and G-quadruplex RNA structures; and finally it has DNA G-quadruplex unfolding activity (12,18). It has been proposed that UP1 unfolds DNA G-quadruplex by binding and subsequently destabilizing the G-quadruplex (12,13,18–22). The UP1 domain has been studied for its structure and interaction with the single-stranded DNA and RNA sequences in detail using X-ray and NMR spectroscopy methods (23–27). Overall these results have proved that UP1 interacts and forms stable complexes with single-stranded DNA/RNA sequences with high affinities.

G-quadruplex structure/conformation dependent binding has been observed for a number of proteins (28–30). hnRNPA1 and TRF2 were shown to preferentially bind the G-quadruplex form of telomere RNA repeats (28–30). Recently, in case of hnRNPA1, the interaction was found to be specific to the bases in the loop nucleotides of the RNA G-quadruplex (31). However, the precise contribution of the UP1 and RGG domains towards this specificity in DNA/RNA G-quadruplexes has not been defined.

The RGG-box region in the CTD of hnRNPA1 has been reported to have both protein and nucleic acid binding capabilities (11,32,33). The RGG-box was shown to help the N-terminal UP1 domain in binding the RNA and DNA sequences cooperatively and has strand annealing activity (33–35). The RGG-box region of hnRNPA1 was reported to undergo methylation on three arginine residues and the F/G-G-G-R-G-G-G-G/F sequence was found to be the preferred recognition motif for arginine methytransferases (36). In recent independent studies, the methylation of the arginine residues in the RGG-box region of hnRNPA1 was shown to be critical for the cytoplasmic activities of hnRNPA1 (37,38). These include the suppression of hnRNPA1’s IRES trans-acting factor activity and stress granule formation and localization by hnRNPA1. The entire CTD after UP1 that includes the RGG-box consists of low complexity sequences and consequently has been postulated to be intrinsically disordered in solution. It is clear that the RGG-box region can differentially modulate the nucleic acid binding properties of UP1 in hnRNPA1, however the direct evidences and the mechanisms are unknown. This work provides a detailed biophysical examination of the recognition of human telomeric DNA G-quadruplexes by the RGG-box of hnRNPA1 and its influence on the G-quadruplex unfolding abilities of UP1.

We have purified UP1 with the RGG-box (UP1+RGG) and the RGG-box alone and have shown that the RGG-box containing region is intrinsically disordered in solution. We have shown that the RGG-box interacts specifically with the G-quadruplex form of telomeric DNA while it does not interact with the ssDNA. NMR chemical shift perturbation results have shown that the RGG-box recognizes the loop nucleotides in the DNA G-quadruplexes and has provided the molecular details of the interaction. Further using ITC, NMR, CD, and fluorescence spectroscopy methods we have shown that the RGG-box helps in specific recognition and enhances the ability of adjacent UP1 to unfold the telomere DNA G-quadruplex.

## MATERIALS AND METHODS

### Cloning, protein expression and purification of UP1, UP1+RGG, and RGG-box

The DNA sequences coding for UP1 (residues 1–196) and UP1+RGG (residues 1–249) of hnRNPA1 (Uniprot identifier P09651-2) were PCR amplified using pET9d-hnRNPA1 as template and sub cloned in to *Escherichia coli* expression vector pET28a between NheI and XhoI restriction sites. Both the constructs contain a C-terminal hexa-histidine (6X-His) affinity purification tag. The RGG-box (residues 197–249) was PCR amplified and sub cloned in to pGEX-6P-1 vector between BamHI and XhoI restriction sites. A TEV cleavage site was introduced to cleave the GST tag from the RGG-box after purification of the protein.

All the proteins were expressed in BL21(DE3) Rosetta cells in LB media. For isotopic labeling of the proteins, a modified M9 minimal media containing ^15^NH_4_Cl and/or ^13^C D-glucose was used. For specific unlabeling, 0.13 mg/l phenylalanine and 0.17 mg/l of tyrosine was added to the minimal media at the time of induction. In case of UP1 and UP1+RGG, bacterial cells were grown at 37°C till the OD_600_ reached 0.6. The temperature was reduced to 30°C and the cells were induced for protein expression with 0.5 mM IPTG and incubated for overnight. For the RGG-box, the cells were incubated at 37°C for 5 h after induction. The cells were harvested by centrifugation at 7000 rpm for 10 min.

#### Purification of UP1 and UP1+RGG

The cells were re-suspended in lysis buffer (50 mM Tris, 500 mM NaCl, 10% glycerol, pH 8 at 25°C) and lysed by sonication. Cell lysates were centrifuged for 1 h at 13000 rpm. For UP1+RGG, 0.1% PEI precipitation was performed by adding PEI from 10% stock drop wise while stirring the lysate at 4°C for 30 min. The lysate was centrifuged at 13000 rpm for 30 min and loaded on a Ni^2+^-NTA column. The proteins of interest were eluted using elution buffer containing 250 mM imidazole (lysis buffer + 250 mM imidazole). Fractions containing protein of interest were concentrated and the proteins were further purified by cation exchange chromatography followed by size exclusion chromatography (SEC) using a S75 column (GE) (Supplementary Figure S1A).

#### Purification of RGG-box

Glutathione-sepharose column was used for the purification of RGG-box. Elution of the GST-RGG-box was performed using elution buffer (50 mM Tris-HCl, 500 mM NaCl, 5 mM βME, 0.02% sodium azide and 10 mM reduced glutathione, pH 8). The protein was dialyzed in 50 mM Tris–Cl, 0.5 mM EDTA, 1 mM DTT and 300 mM NaCl, pH 8, followed by the GST tag removal by TEV cleavage. For the cleavage, TEV protease was added to the protein (at 1:500 (w/w) ratio). The cleavage reaction was incubated at 16°C for overnight. The mixture was then loaded on SEC S75 column for the RGG-box purification. The final purity of the proteins was ascertained using SDS-PAGE and MALDI-MS analyses and adjudged to be >95% purified (Supplementary Figure S1B and C).

### DNA preparation for CD, NMR and fluorescence kinetics experiments

The DNA sequences (Table 1) for binding studies with UP1, UP1+RGG and the RGG-box were ordered from Eurofins except the abasicloops-Tel22 DNA that was ordered from Dharmacon. For fluorescence studies, 5′-FLUO-(Tel22)-TAMRA-3′ (F-Tel22-T) [where fluorescein (FLUO) and tetramethylrhodamine (TAMRA) are fluorescent dyes] were synthesized by Sigma Aldrich. DNA samples were prepared in 100 mM NaCl/KCl containing buffer for the formation of G-quadruplex. The buffer composition was 20 mM Tris-Cl (pH 8), 100 mM NaCl/KCl for CD and ITC experiments and 10 mM sodium phosphate/potassium phosphate (pH 6.5) with 100 mM NaCl/KCl for NMR experiments. The DNA samples were heated at 95°C for 3 min and then cooled on ice for 30 min. In case of Tel12, Tel6 and abasicloops-Tel22 DNA the samples were incubated at 4°C for overnight for G-quadruplex to form. The concentrations of the DNAs were calculated by ultraviolet (UV) absorbance at 260 nm using a UV spectrophotometer (Eppendorf BioSpectrometer) and the molar extinction coefficients, which were calculated based on the sequence of the particular DNA. The formation of G-quadruplex by Tel22 was confirmed using CD and NMR spectroscopy.

**Table 1.** DNA sequences used in this study

DNA	Sequence
Tel22ss	d[AG_2_A(T_2_AG_2_A)_3_]
Tel22	d[AG_3_(T_2_AG_3_)_3_]
Tel6	d[T_2_AG_2_A]
Tel12	d[(T_2_AG_2_A)_2_]
abasicloops-Tel22	d[A(G)_3_(-/dSpacer//dSpacer//dSpacer/-G _3_)_3_] where /dSpacer/ is abasic nucleotide modification.

### ITC experiment

Isothermal titration calorimetry (ITC) experiments were performed using iTC200 instrument (GE) at 25°C. Samples were thoroughly degassed before the experiment. For the titrations, the sample cell was filled with 5 μM of the DNA and titrated with 100–200 μM of the protein. 20 injections of the titrant were performed at an interval of 3 min between each injection. The heat of dilution was subtracted from the integrated heat data and the data was fit for one site binding model using ORIGIN software provided by the vendor (GE). All the parameters were kept floating during the data fitting.

### CD spectroscopy

The structural changes in G-quadruplex (Tel22) when titrated with UP1, UP1+RGG, TriRGG-mutant, and AllRGG-mutant were monitored using CD spectroscopy. CD titrations were carried out on a JASCO J-715 spectropolarimeter. The titration experiments were performed in 1 cm path length cuvette (Hellma Analytics) at 25°C at a scanning speed of 100 nm/min with a response time of 4 s. The wavelength scan was done from 225 nm to 325 nm. 5 μM of the DNA was titrated with increasing protein concentration. The average of three spectral scans was taken for each sample followed by baseline correction to negate the contribution from the buffer. The data was normalized to molar ellipticity per residue.

### NMR spectroscopy

1D ^1^H NMR and 2D ^15^N–^1^H HSQC spectra of protein and 1D ^1^H NMR spectra of DNA were recorded on Bruker 700 MHz, equipped with a cryoprobe, at 298K. ^15^N and ^13^C uniformly double-labeled sample for isolated RGG-box was prepared for the backbone assignment. Standard 3D triple resonance HNCACB and CBCA(CO)NH spectra were recorded on a Varian 600 MHz spectrometer with a cryoprobe (39,40). The 1D ^1^H experiments were recorded using excitation-sculpting scheme for water suppression. 550 μl of 280 μM of the DNA sample was prepared in 10 mM sodium phosphate (pH 6.5) and 100 mM NaCl/KCl respectively. 10% D_2_O was added to the sample for the spectrometer deuterium lock. The DNA was titrated with increasing concentration of UP1 and UP1+RGG (in both Na^+^ and K^+^ buffer).

The chemical shift changes were monitored by titrating ^15^N labeled RGG-box (153 μM) with increasing concentration of DNA (Tel22, Tel12 and Tel6) followed by 10 min of incubation and recording a 2D ^15^N–^1^H HSQC spectrum of protein at each step of titration. NMR spectra were recorded at 298K on a Bruker 700 MHz NMR spectrometer at 298K. The NMR data was processed using Bruker TOPSPIN 3.1 or NMRPipe and analyzed using NMRFAM-SPARKY (41,42).

For abasicloops-Tel22 DNA titration, we used lower concentration of ^15^N labeled RGG-box (110 μM) due to limited availability of abasicloops-Tel22 DNA. For this concentration of protein, we could achieve a final 1:1 molar ratio of DNA:protein, but it resulted in the relatively low signal to noise ratio in the 2D ^15^N–^1^H HSQC spectrum of protein. However, the chemical shift perturbations, in fast exchange, observed were distinct and could be followed unambiguously.

### NMR chemical shift perturbation

The observed chemical shift changes for the RGG-box—Tel22 titrations were calculated using the formula ${\rm{\Delta }}{\delta _{obs}} = {[ {{{( {{\rm{\Delta }}{\delta _H}} )}^2} + {{( {{\rm{\Delta }}{\delta _N}/5} )}^2}} ]^{1/2}}$, where the chemical shift changes in the ^1^H and ^15^N dimensions are denoted by ${\rm{\Delta }}{\delta _H}$ and ${\rm{\Delta }}{\delta _N}$ respectively. The observed chemical shift changes, as a function of the DNA concentration added to the protein sample at each titration step, were then individually fit to obtain apparent *K*_d_s for each residue (local independent fit) (43). The following formula (implemented in Origin 9.0) was used for calculating the apparent dissociation constant (*K*_d_):

\begin{eqnarray*}\ {\rm{\Delta }}{\delta _{obs}} = {\rm{\Delta }}{\delta _{max}}\ \frac{{\left[ {\left( {\left[ {P{]_T} + \left[ {\rm{D}} \right] + {K_d}} \right) - \sqrt {({{\left[ {P{]_T} + \left[ {\rm{D}} \right] + {K_d}} \right)}^2} - (4{{\left[ P \right]}_T}\left[ D \right]} } \right)} \right]}}{{2{{\left[ P \right]}_T}}},\end{eqnarray*}

where $\ {\rm{\Delta }}{\delta _{obs}}$ is the observed chemical shift changes at a particular concentration of DNA added, ${\rm{\Delta }}{\delta _{max}}$ is the total chemical shift change at free and fully saturated states, ${[ P ]_T}$ is the total protein concentration used for the titration experiment and $[ {\rm{D}} ]$ is the concentration of the DNA added at each step in the titration.The *K*_d_s for each residue were plotted against the ratio of molar concentration of DNA added to protein and the ${\rm{\Delta }}{\delta _{obs}}$ at each titration step. Fourteen residues that had individual fit *K*_d_s in the 150.76 ± 7.31 μM to 432.86 ± 15.56 μM range were globally fit to a shared *K*_d_ of 349 ± 35 μM.

### Fluorescence kinetics

The labeled FAM-Tel22-TAMRA (F-Tel22-T) was excited at 470 nm and the emission maximum was at 516 nm on recording the spectrum between 490–650 nm. 100 nM of F-Tel22-T in 20 mM Tris-Cl, pH 8 and 100 mM KCl was mixed with 4× molar excess of UP1 (in K^+^ and Na^+^) and UP1+RGG (in K^+^ and Na^+^) and fluorescence kinetics was measured. The spectra were recorded on JASCO FP-6300 spectrofluorometer at 25°C. Fluorescence intensity at 516 nm was recorded as a function of time at a regular internal of 10 s using band slits of 5 nm.

## RESULTS

### The RGG-box containing region in hnRNPA1 is intrinsically disordered

UP1+RGG (residues 1–249), UP1 (residues 1 to 196), and RGG-box (residues 197–249) were purified using standard purification methods (see Materials and Methods) (Figure 1A). While UP1 and UP1+RGG were C-terminally hexa-histidine tagged, the RGG-box region was purified as an N-terminally GST (glutathione-S-transferase) tagged protein. The GST tag was cleaved using TEV protease from the fusion protein to yield the purified isolated RGG-box.

The identity and purity of the samples were ascertained using SDS-PAGE analysis and mass-spectrometry analysis of the purified proteins (Supplementary Figure S1A–C). 2D ^15^N–^1^H HSQC NMR spectrum of UP1 showed that the purified protein was folded (Figure 1B). The spectrum of UP1 matched with the published spectrum of UP1 (26). The 2D ^15^N–^1^H HSQC NMR spectrum of UP1+RGG overlaid well with the spectrum of UP1 with extra cross-peaks arising from the RGG-box region clustered in the center of the spectrum (∼8.3 ppm in proton dimension), which is characteristic of an unfolded/disordered protein indicating that the RGG-box region in UP1+RGG is disordered in solution (Figure 1B).

**Figure 2.**
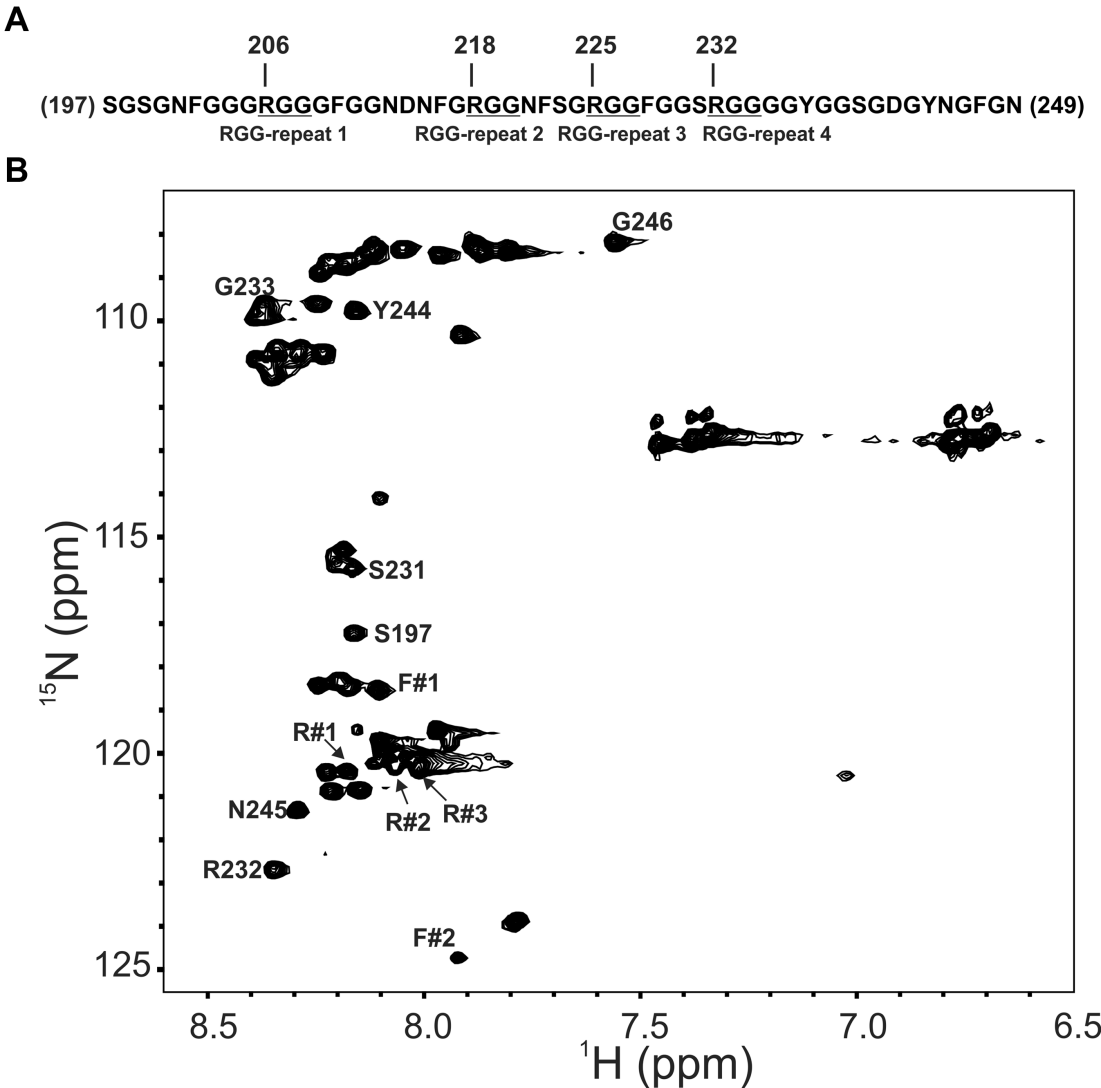
Characterization of the RGG-box using NMR spectroscopy. (**A**) The sequence of the hnRNPA1 RGG-box used in this study. The four RGG-repeats are mentioned and the ‘RGG’ sequences are underlined. (**B**) The partially assigned 2D ^15^N–^1^H HSQC spectrum of the intrinsically disordered RGG-box. The unambiguously assigned and identified residues are marked.

We prepared ^15^N uniformly labeled isolated RGG-box and recorded a 2D ^15^N–^1^H HSQC NMR spectrum. The cross peaks of the isolated RGG-box matched well with the RGG region of UP1+RGG spectrum (Figure 1B). A dispersion of only ∼1 ppm in proton dimension and sharp line widths of the cross peaks revealed that the isolated RGG-box is intrinsically disordered in solution. We observed a total of 47 cross peaks out of expected 53 NHs from the backbone in the RGG-box sequence (Figure 2A and B).

The RGG-box contains 28 glycine residues out of a total of 53 residues in its sequence (53%). Combined with this, the repetitive nature of the RGG-repeats in the sequence made it challenging to unambiguously assign resonances in the RGG-box (Figure 2A). However, upon careful analysis of the triple resonance NMR spectra: CACB(CO)NH/HNCACB, we could confirm the assignment of some of the residues including assignment of R232 from the fourth RGG-repeat (Figure 2B and Supplementary Figure S2A). We could also locate other three arginine residues (R206, R218 and R225) in the 2D ^15^N–^1^H HSQC spectrum (Supplementary Figure S2B), however the sequential assignment of these arginine residues could not be ascertained unambiguously due to the repetitive nature of ‘GRGG’ stretches in which these arginine residues are located. These arginine residues are referred as R#1, R#2, and R#3 in the text and figures. Apart from the glycine and arginine residues, aromatic residues phenylalanine and tyrosine have been shown to interact with the nucleic acid substrates in RGG-box in other protein (44,45). To locate and assign the tyrosine and phenylalanine residues in the RGG-box of hnRNPA1, we used the strategy of selective unlabeling of the protein for these residues. In this strategy, we used unlabeled amino acid in the ^15^N labeled media during protein induction by IPTG. This results in preferential incorporation of added unlabeled amino acid while the rest of the amino acids are uniformly labeled. However, the low water solubility of these two amino acids limits the availability of free unlabeled amino acid for preferential uptake resulting in inefficient incorporation. Nonetheless we could locate one tyrosine (out of two) and two phenylalanine (out of six) residues in the 2D ^15^N–^1^H HSQC spectrum of RGG-box (Figure 2B and Supplementary Figure S3A–C).

This partial assignment and identification of the residues in the 2D ^15^N–^1^H HSQC spectrum of the RGG-box provided us with the list of residues that could act as molecular probe to understand the interaction of RGG-box with DNA (Figure 2A). The unambiguously assigned residues in the 2D ^15^N–^1^H HSQC NMR spectrum of RGG-box are: S197, S231, R232, G233, Y244, N245 and G246 (Figure 2B).

### RGG-box shows weak but structure specific interaction with the telomere G-quadruplex DNA

Table 1 shows the DNA sequences used in this study. While the Tel22ss sequence is designed to exist in the single stranded conformation, other DNA sequences adopt G-quadruplex structures of different topologies in the presence of monovalent metal ions such as Na^+^ or K^+^ (Supplementary Figure S4A–F). In Tel22ss sequence, the third guanine of TTAGGG repeat is mutated to adenine in all the four repeats. This prevents the formation of any G-tetrad and thereby hinders the ability of the sequence to form G-quadruplex. Therefore Tel22ss sequence is used as a single-strand DNA control. The CD spectrum of Tel22ss in the presence of either 100 mM NaCl or 100 mM KCl revealed that it does not adopt a quadruplex structure and remains in single-stranded conformation in solution with no positive ellipticity at 295 nm (Supplementary Figure S4G). The 1D ^1^H spectrum of Tel22ss showed no peak in the imino proton region, again confirming that it remains in the single stranded conformation in solution (Supplementary Figure S5A). In the G-quadruplex forms, the three G-quartets are stacked on each other with the help of a monovalent cation. The Tel22 sequence contains four repeats of guanine and forms unimolecular G-quadruplex structure with three loops (Supplementary Figure S4B and C) (46). Tel22 adopts ‘mixed-parallel/antiparallel basket type’ conformation in the presence of Na^+^ salt. The same DNA adopts ‘mixed-parallel/antiparallel hybrid-type-1’ and ‘mixed-parallel/antiparallel hybrid-type-2’ conformations in the presence of physiologically relevant intracellular concentration of K^+^ ion (> 100 mM), which are at equilibrium with each other (46).

We confirmed the formation of G-quadruplex structures by Tel22 in the presence of 100 mM NaCl and 100 mM KCl by CD and NMR spectroscopy (Supplementary Figure S4G and H, and Supplementary Figure S5B and C). These results are in agreement with the previously published spectra of this DNA (18,47).

There is no detailed biophysical study that showed the direct role of the RGG-box of hnRNPA1 in telomeric DNA recognition and G-quadruplex unfolding. We probed interaction of the RGG-box with the Tel22ss and Tel22 DNA using ITC. However no appreciable enthalpy change was observed and therefore we could not ascertain the equilibrium dissociation constant and other thermodynamic parameters for these interactions. We postulated that the interaction of the RGG-box with the DNA might be weak in nature. Therefore, we used NMR spectroscopy to detect the interaction of the isolated RGG-box with the telomeric DNA. NMR chemical shifts are extremely sensitive to the chemical environment and therefore even weak interactions can be detected using NMR.

**Figure 3.**
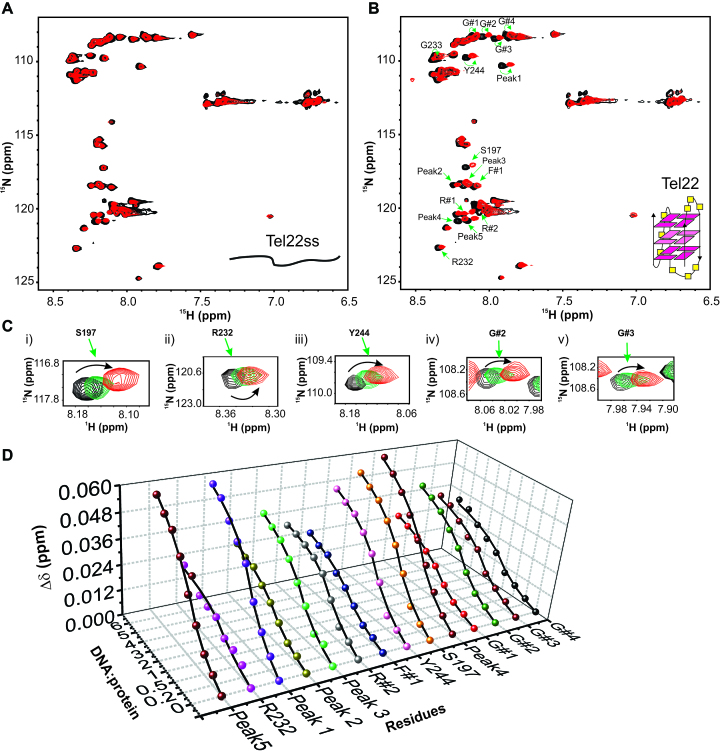
Interaction of RGG-box with the single stranded and G-quadruplex DNA monitored through NMR spectroscopy. (**A**) 2D ^15^N–^1^H HSQC spectrum of the free RGG-box (black) and in complex with Tel22ss at 1:6 protein to DNA molar ratio (red). No significant chemical shift perturbations were observed for this interaction. Single stranded Tel22ss is shown as a cartoon. (**B**) 2D ^15^N–^1^H HSQC spectrum of the RGG-box (black) and in complex with Tel22 at 1:6 protein to DNA molar ratio (red). Specific chemical shift perturbations were observed for several residues (marked with green arrows). A representative cartoon of monomeric G-quadruplex form of Tel22 is shown (only one conformation in K^+^ ion is shown). (**C**) A subset of residues of RGG-box that show specific chemical shift perturbation upon addition of Tel22 is shown. The RGG-box and Tel22 complex was in fast exchange (weak binding) as we observed continuous movement of resonance peaks upon addition of increasing amount of the Tel22 DNA. Three steps of titration at different protein to DNA ratios are shown: black at 1:0, green at 1: 1, and red at 1:6. (**D**) The titration curves showing chemical shift change plotted as a function of increasing DNA:protein ratio for 14 interacting residues of the RGG-box is shown.

We titrated ^15^N uniformly labeled RGG-box with increasing amount of single stranded Tel22ss DNA and recorded 2D ^15^N–^1^H HSQC spectrum at each step of titration. No chemical shift perturbation was observed even at 1:6 molar ratio of protein to DNA (Figure 3A). This clearly showed that the isolated RGG-box region does not interact with the single stranded DNA. Next, we titrated the ^15^N uniformly labeled RGG-box with increasing concentration of G-quadruplex Tel22 DNA and recorded 2D ^15^N–^1^H HSQC spectrum at each step of titration. Remarkably, we observed distinct and specific chemical shift perturbations for a selected number of resonances in the RGG-box spectrum (Figure 3B). The G-quadruplex Tel22 and RGG-box complex was in fast exchange regime on NMR timescale indicating a weak but specific interaction (Figure 3Ci–v). This unambiguously showed that the RGG-box specifically recognizes the G-quadruplex form of DNA. We observed distinct chemical shift perturbations (CSP) in fast exchange time regime for a number of residues (∼17 residues) including three arginine (R232, R#1, and R#2) and four glycine (G#1 to G#4) residues from RGG-motif in our titration (Figure 3B and C). R232 and R#2 showed relatively significant CSPs, while R#1 showed weak CSP (Supplementary Figure S6A and B). R#3 did not show any significant chemical shift perturbation. Apart from this, other assigned residues that showed significant CSPs include: F#1, S197 and Y244. A number of other unassigned peaks were also perturbed upon Tel22 binding and are marked in Figure 3B (also see Supplementary Figure S7A and Supplementary Table S1).

Fitting the chemical shift changes of 14 residues as a function of DNA concentration gave values of the apparent dissociation constant (*K*_d_) for the individual fits (ranging from ∼151 to 433 μM) (please see Materials and Methods section, Figure 3D, and Supplementary Figure S7B). All the individual fit *K*_d_s were globally fit to an apparent *K*_d_ of 349 ± 35 μM (Supplementary Table S1).

### The interaction of the RGG-box with the telomeric G-quadruplex DNA is dependent on the loop nucleotides

Telomere DNA repeats of 6mer result in a tetrameric parallel G-quadruplex devoid of any loop, whereas 12mer telomere repeats result in a parallel dimeric structure with two loops in the presence of 100 mM KCl (Supplementary Figure S4D and E). We confirmed the formation of G-quadruplex structures with distinct topologies by CD and NMR spectroscopy (Supplementary Figures S4I and S5D and E).

**Figure 4.**
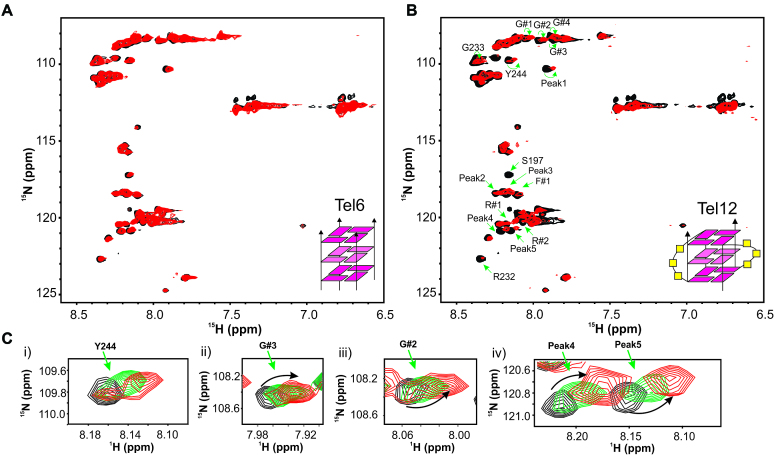
Loop dependent recognition of G-quadruplex structure by the RGG-box. (**A**) 2D ^15^N–^1^H HSQC spectrum of the free RGG-box (black) and in complex with Tel6 at 1:6 protein to DNA molar ratio (red). No significant chemical shift perturbations were observed for this interaction. Tetrameric G-quadruplex form of Tel6 that is devoid of loops is shown as a cartoon. (**B**) 2D ^15^N–^1^H HSQC spectrum of the free RGG-box (black) and in complex with Tel12 at 1:1 protein to DNA molar ratio (red). Dimeric G-quadruplex form of Tel12 consisting of two loops is shown as a cartoon. Specific chemical shift perturbations were observed for several residues (marked with green arrows). (**C**) A subset of residues of RGG-box that show specific chemical shift perturbation upon addition of Tel12 is shown. The RGG-box and Tel12 complex was in fast to intermediate exchange. Three steps of titration at different protein to DNA ratios are shown: black at 1:0, green at 1:0.5 and red at 1:1.

Titration of ^15^N uniformly labeled RGG-box was carried out with increasing amount of tetrameric (6mer) and dimeric (12mer) G-quadruplex DNAs. Interestingly, we observed no significant chemical shift perturbation in 2D ^15^N–^1^H HSQC spectra of the RGG-box when titrated with the 6mer G-quadruplex DNA that is devoid of loops (Figure 4A). However, when titrated with the 12mer dimeric G-quadruplex DNA, we observed specific chemical shift perturbations in the 2D ^15^N–^1^H HSQC spectra similar to the monomeric Tel22 G-quadruplex DNA (Figure 4B and C). Progressive broadening of resonance peaks in 2D ^15^N–^1^H HSQC spectra were observed upon titrating the protein with excess DNA (above 1:1 protein to DNA), indicating that the complex is likely in the fast to intermediate-exchange regime in NMR time scale (data not shown). Peaks that showed either perturbation and/or broadening overlapped with the resonances that showed perturbation in case of Tel22 titration. This included chemical shift perturbation of G#1-4, R232 and R#2 and weak perturbation in R#1 (Figure 4B and Supplementary Figure S6B). Taken together these results clearly demonstrated that the interaction of RGG-box is highly specific for the structured loop residues of the DNA G-quadruplex (in Tel22 and Tel12), whereas it does not recognize either the single stranded DNA (Tel22ss) or G-quadruplex that lacks loops (Tel6).

**Figure 5.**
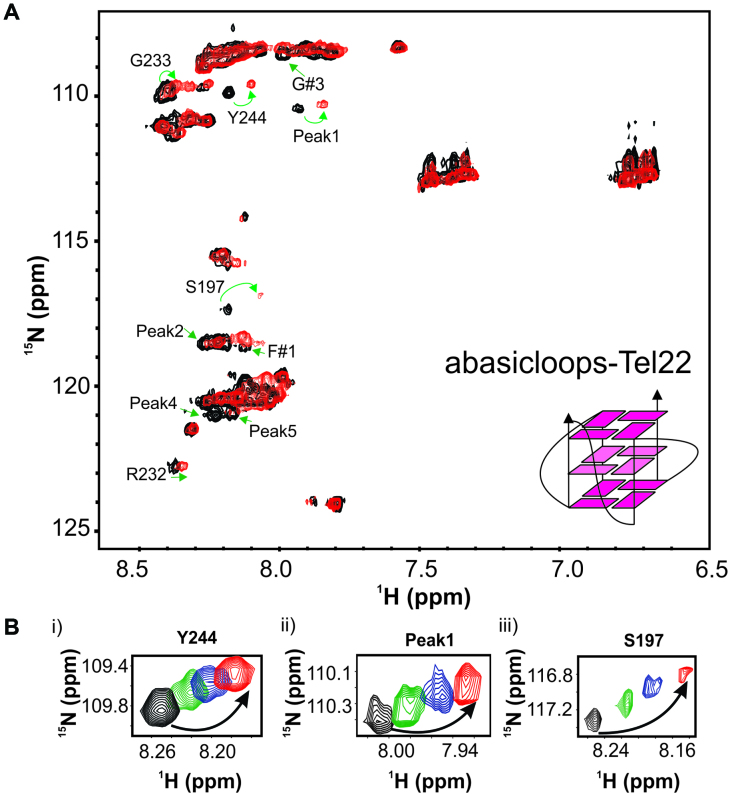
The RGG-box interacts with the G-quadruplex with abasic deoxyribose loops. (**A**) 2D ^15^N–^1^H HSQC spectrum of the free RGG-box (black) and in complex with abasicloops-Tel22 DNA at 1:1 protein to DNA molar ratio (red). A representative cartoon of monomeric G-quadruplex form of abasicloops-Tel22 is shown. Specific chemical shift perturbations were observed for several residues (marked with green arrows). (**B**) A subset of residues of the RGG-box that show specific chemical shift perturbation upon addition of abasicloops-Tel22 is shown. The RGG-box and abasicloops-Tel22 complex was in fast exchange. Four steps of titrations at different protein to DNA ratios are shown: black at 1:0, green at 1:0.2, blue at 1:0.5 and red at 1:1.

To gain insight into whether the interaction of the RGG-box with the loop nucleotides is specific for bases or the sugar-phosphate or both, we designed and ordered a variant of Tel22 sequence, where the loop residues contain abasic deoxyribose (called abasicloops-Tel22) (Table 1 and Supplementary Figure S4F). We confirmed the formation of parallel G-quadruplex by abasicloops-Tel22 by CD and NMR spectroscopy (Supplementary Figure S4I and Supplementary Figure S5F). ^15^N uniformly labeled RGG-box was titrated with increasing amount of abasicloops-Tel22 and a 2D ^15^N–^1^H HSQC spectrum was recorded at each titration step (please see Materials and Method section for details). We observed distinct fast exchange chemical shift perturbations for a subset of residues that were perturbed upon Tel22 and Tel12 interaction (Figure 5A and B). This unambiguously showed that the abasicloops-Tel22 interacts with the RGG-box. The residues in RGG-box that showed distinct chemical shift perturbation are: S197, R323, G233, Y244, S197, G#3, and Peak 1, peak 2, peak 4 and peak 5. Taken together, the results show that likely both sugar-phosphate and bases in the loop nucleotides are involved in G-quadruplex recognition and binding.

### UP1+RGG interacts with single stranded and G-quadruplex telomeric DNA

**Figure 6.**
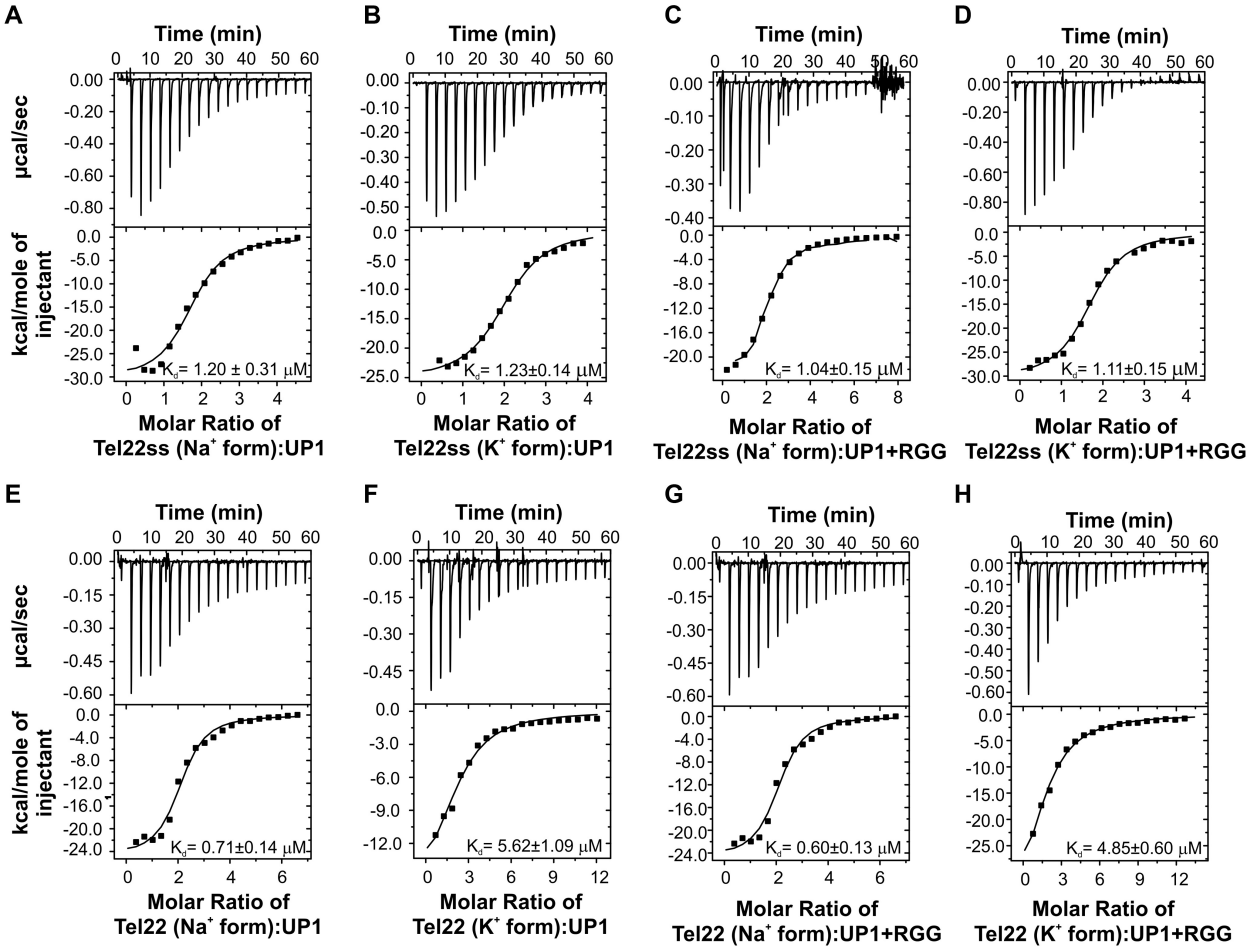
Interaction of UP1+RGG and UP1 with the single stranded and G-quadruplex DNA monitored through ITC. Raw and fitted isotherms are shown and the equilibrium K_d_s obtained upon fitting of the raw data is mentioned in each panel. (**A, B**) Interaction of UP1 with the single stranded Tel22ss DNA in the presence of 100 mM NaCl and 100 mM KCl respectively. (**C, D**) Interaction of UP1+RGG with the single stranded Tel22ss DNA in the presence of 100 mM NaCl and 100 mM KCl respectively. (**E, F**) Interaction of UP1 with the Na^+^ and K^+^ forms of Tel22 G-quadruplex DNA respectively. (**G, H**) Interaction of UP1+RGG with the Na^+^ and K^+^ forms of Tel22 G-quadruplex DNA respectively.

To compare the effect of the RGG-box on DNA binding functions of the UP1 domain, we carried out thermodynamic analysis of UP1 and UP1+RGG using ITC. The titration of Tel22 and Tel22ss with UP1 and UP1+RGG were performed separately in the presence of 100 mM NaCl and 100 mM KCl in buffer. For all the experiments, DNA solution in the cell was titrated with either UP1 or UP1+RGG proteins. Figure 6A–H shows the raw and fitted ITC isotherms and Table 2 summarizes the results obtained for each titration. All the interactions are enthalpically driven and we observed a stoichiometry (*n*) of ∼2 for all the interactions, which is in agreement with the previous observations (18).

**Table 2.** Thermodynamic parameters derived from ITC experiments for the titration of UP1 and UP1+RGG into telomere single-stranded and G-quadruplex DNA

S.No	Experiment	Salt (100 mM)	*K*_d_ (μM)	Δ*G* (kcal/mol)	Δ*H* (kcal/mol)	*T*Δ*S* (kcal/mol)	*n*
1	UP1–Tel22ss	NaCl	1.20 ± 0.31	-8.07 ± 2.05	−30.48 ± 1.428	−22.41 ± 3.478	1.73 ± 0.06
2	UP1–Tel22ss	KCl	1.23 ± 0.14	−8.05 ± 0.9	−25.32 ± 0.52	−17.27 ± 1.42	2.04 ± 0.03
3	UP1+RGG–Tel22ss	NaCl	1.04 ± 0.15	−8.15 ± 1.14	−24.65 ± 0.84	−16.50 ± 1.98	1.98 ± 0.05
4	UP1+RGG – Tel22ss	KCl	1.11 ± 0.15	−8.11 ± 1.08	−30.54 ± 0.7	−22.43 ± 1.79	1.70 ± 0.03
5	UP1–Tel22	NaCl	0.71 ± 0.14	−8.38 ± 1.68	−14.26 ± 0.37	−5.88 ± 2.05	2.08 ± 0.04
6	UP1–Tel22	KCl	5.62 ± 1.09	−7.15 ± 1.39	−17.84 ± 2.012	−10.69 ± 3.402	2.21 ± 0.20
7	UP1+RGG–Tel22	NaCl	0.60 ± 0.13	−8.48 ± 1.81	−24.84 ± 0.907	−16.36 ± 2.717	2.05 ± 0.06
8	UP1+RGG–Tel22	KCl	4.85 ± 0.60	−7.42 ± 0.88	−44.89 ± 4.84	−37.65 ± 5.72	1.72 ± 0.15

UP1 interacts with single stranded Tel22ss formed in 100 mM NaCl and 100 mM KCl with *K*_d_s of 1.20 ± 0.31 and 1.23 ± 0.14 μM respectively (Figure 6A and B and Table 2). Since the Tel22ss sequence remains in the single stranded conformation in both 100 mM NaCl and 100 mM KCl solutions, it is not surprising that the *K*_d_s are similar in both the cases. Next, the Tel22ss was titrated with UP1+RGG in the presence of 100 mM NaCl and 100 mM KCl and similar *K*_d_s of 1.04 ± 0.15 μM and 1.11 ± 0.15 μM were determined respectively (Figure 6C and D and Table 2). The dissociation constants for Tel22ss interaction with UP1 and UP1+RGG are almost identical, suggesting that the ‘extra’ RGG-box in UP1+RGG does not contribute significantly towards binding of UP1 to the single-stranded DNA. This corroborates our observation that the isolated RGG-box does not bind to the single stranded Tel22 sequence in our NMR titrations (Figure 3A).

In a previous study, Tel22 G-quadruplex recognition and destabilization by UP1 was characterized by ITC and CD spectroscopy titrations (18). The complex ITC isotherm obtained in that study, for UP1 and Na^+^ form of Tel22 interaction was suggestive of multiple events and was fitted to three events of binding. The isotherm obtained for the K^+^ form of Tel22 and UP1 interaction was distinct and was fitted to two events of binding (18). In our titrations, though we do have hints of initial binding events present at lower concentration of UP1, we could however; best fit our data with the one event binding. We carried out our titrations on iTC200 machine (GE) that uses less volume of protein and DNA compared to the VP-ITC that was used in the previous study. The larger volume allows more titrations steps and therefore may have led to the detection of initial steps of binding and unfolding of G-quadruplexes (18). However, a simpler binding model and data fitting allowed us to compare the interactions of UP1 and UP1+RGG with G-quadruplex DNA directly as described next. The final fitted ITC data presented here, therefore likely reflects the average of all the events occurring during the experimental run.

We carried out the titration of UP1 and UP1+RGG with both Na^+^ and K^+^ forms of Tel22 G-quadruplex DNA. In our one event-binding model, we report *K*_d_s of 0.71 ± 0.14 μM and 5.62 ± 1.09 μM for UP1 interaction with Na^+^ and K^+^ form of Tel22 respectively (Figure 6E and F and Table 2). For UP1+RGG interaction with Tel22 the *K*_d_ values are 0.60 ± 0.13 μM and 4.85 ± 0.60 μM for the Na^+^ and K^+^ forms of G-quadruplexes respectively (Figure 6G and H and Table 2). As evident from these results, UP1 and UP1+RGG interact with Tel22 G-quadruplexes with similar affinities with a small decrease in K_d_s for UP1+RGG. Interestingly, however, binding of UP1+RGG with Tel22 proceeds with larger enthalpy change (Δ*H*) of -44.89 kcal/mol compared to the interaction of UP1 with K^+^ form of Tel22 G-quadruplex (–17.84 kcal/mol). A similar decrease in Δ*H* was observed for the Na^+^ form of Tel22 and UP1+RGG interaction (Table 2). There is consequently a larger entropy change (a negative *T*Δ*S* term) suggesting a more ordered initial complex formation in case of UP1+RGG with the G-quadruplex (Tel22). In general, the specific protein–DNA/RNA interactions proceed with comparatively large enthalpy change (48). Therefore, our results suggest that the UP1+RGG interaction with the G-quadruplex DNA results in the formation of a more specific protein-DNA complex compared to UP1 alone.

### Unfolding of telomeric G-quadruplexes by UP1+RGG

CD and NMR spectroscopy are excellent methods to probe the structural changes in G-quadruplex and monitor its unfolding (49,50). In the CD spectrum, the Na^+^ form of G-quadruplex structure is characterized by strong positive maxima at 295 and 245 nm and a minima at 265 nm. In contrast, the same Tel22 G-quadruplex prepared in the presence of K^+^ ions shows a markedly different CD spectrum with a positive ellipticity at 295 nm with a broad shoulder at 265 nm (Supplementary Figure S4G). The structure and dynamics of the loops in the G-quadruplexes are cation dependent (51). There is difference in the loop conformation and G-tetrad stacking between the Na^+^ and K^+^ forms. The loss of the distinct CD signature of the G-quadruplexes in Na^+^ and K^+^ environment upon binding with the ligand or protein would therefore indicate unfolding of the quadruplex structure by the ligand or proteins (18).

The thermodynamic stability of the G-quadruplex structures formed by Tel22 in Na^+^ and K^+^ buffers are also different (18). The changes in the CD signal as a function of temperature were monitored and it revealed different melting temperatures, *T*_m_, of 59°C and 68°C for Tel22 in Na^+^ and K^+^ forms of the G-quadruplex respectively (Supplementary Figure S4H). Tight packing of the G-quartet in case of K^+^ metal ion results in a more stable G-quadruplex structure compared to the Na^+^ form (52).

**Figure 7.**
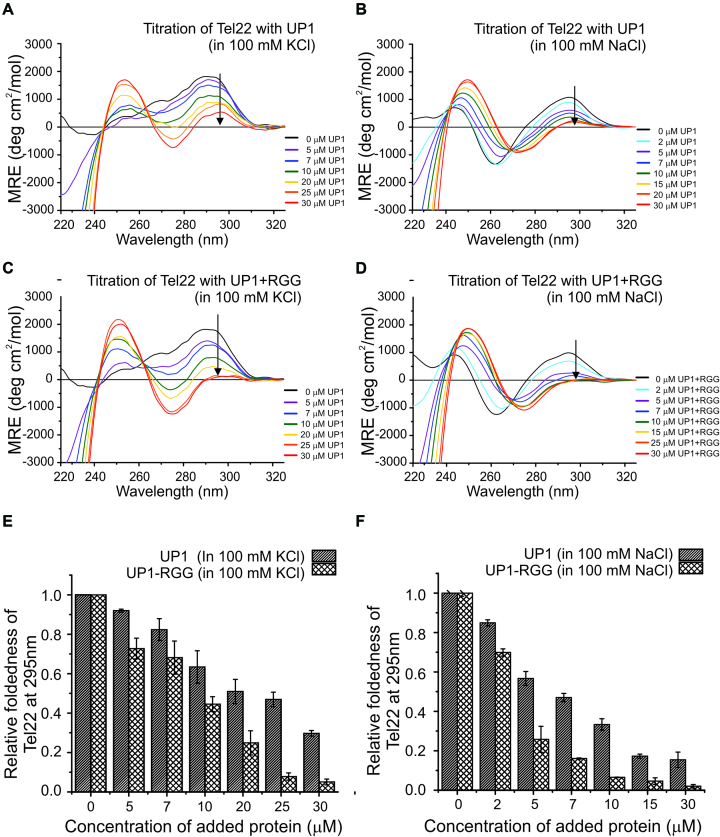
Telomere DNA G-quadruplex unfolding by UP1+RGG and UP1 monitored using CD spectroscopy. (**A, B**) Unfolding of K^+^ and Na^+^ forms of Tel22 G-quadruplex DNA by UP1. The G-quadruplex DNAs were titrated with increasing molar excess of proteins. The black arrow in the spectra indicates the gradual decrease in ellipticity at 295 nm with increasing protein concentration. (**C, D**) Unfolding of K^+^ and Na^+^ forms of Tel22 G-quadruplex DNA by UP1+RGG. The G-quadruplex DNAs were titrated with increasing molar excess of proteins. The black arrow in the spectra indicates the gradual decrease in ellipticity at 295 nm with increasing protein concentration. (**E, F**) The ellipticity at 295 nm was normalized and plotted to show the relative foldedness of both K^+^ and Na^+^ forms of quadruplexes upon UP1 or UP1+RGG addition at each step of titration.

Titration of 5 μM DNA G-quadruplex formed in the presence of 100 mM KCl and NaCl solution by increasing concentration of UP1 and UP1+RGG was carried out and monitored using CD spectroscopy. We observed decrease in ellipticity at 295 nm for both the forms of Tel22 G-quadruplexes with increasing concentration of UP1 and UP1+RGG, which indicates unfolding of the quadruplex structure (Figure 7A–D). We quantitated the G-quadruplex unfolding mediated by UP1 and UP1+RGG by normalizing the CD signal at 295 nm and plotted it against the concentration of protein added. UP1+RGG almost completely unfolds both Na^+^ and K^+^ forms of the G-quadruplexes at a concentration of 10 μM (at molar ratio 1:2 of Tel22:UP1+RGG) and at 25 μM (at a molar ratio of 1:5) respectively (Figure 7E and F). On the contrary, UP1 is unable to completely unfold the K^+^ form of quadruplex even at a concentration of 30 μM (molar ratio of 1:6) (Figure 7E). In case of Na^+^ form of the G-quadruplex, UP1 is able to unfold the G-quadruplex to the point where it still remains 15–20% structured at 15 μM protein added (molar ratio 1:3) and fails to unfold it beyond this even at 1:6 molar ratio of Tel22 to UP1 (Figure 7F). Comparative unfolding of Na^+^ form of G-quadruplex by both UP1 and UP1+RGG is efficient as compared to the K^+^ form of the G-quadruplex. This is likely due to the fact that the K^+^ form of G-quadruplex is more stable structure than the Na^+^ form as described earlier (Supplementary Figure S4H).

**Figure 8.**
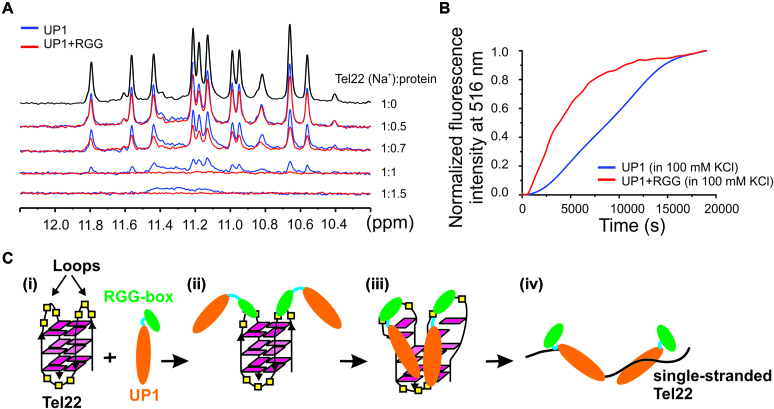
Telomere DNA G-quadruplex unfolding by UP1+RGG and UP1 monitored using NMR and fluorescence spectroscopy. (**A**) 1D ^1^H NMR spectra of Na^+^ form of Tel22 showing gradual loss of imino proton peaks upon titration with increasing concentrations of UP1 (blue) and UP1+RGG (red). (**B**) Unfolding of the 5′-FAM and 3′-TAMRA labeled K^+^ form of Tel22 DNA G-quadruplex (5′FAM-Tel22-TAMRA3′) by UP1 (blue) and UP1+RGG (red) monitored by observing the emission of FAM at 516 nM. 5′FAM-Tel22-TAMRA3′ DNA was mixed with 4 molar equivalents of UP1 or UP1+RGG and the emission spectrum was recorded over a time period. (**C**) Proposed model for RGG-box assisted recognition and unfolding of telomere DNA G-quadruplex unfolding by UP1+RGG.

In the 1D ^1^H NMR spectra, the resonance peaks in the downfield shifted imino region (10–12 ppm) of G-quadruplexes are characteristic of G-quadruplex formation (Supplementary Figure S5B-E) (49). The broadening and disappearance of imino peaks upon ligand or protein binding indicates the unfolding of the G-quadruplex structures. Titration of Tel22 (both Na^+^ and K^+^ forms) with increasing concentration of UP1 and UP1+RGG was performed and the unfolding of the G-quadruplex structures was monitored by the disappearance of the imino proton peaks in 1D ^1^H NMR spectra. The titrations were done at pH 6.5 and at 298K. The proteins were added to the DNA in the molar ratios of 1:0.2, 1:0.4, 1:0.5, 1:0.7, 1:1, 1:1.5 and 1:2. In the titration experiments of Tel22 (in Na^+^) with the proteins, the intensity of the imino protons of DNA reduced rapidly in case of UP1+RGG as compared to UP1 (Figure 8A). At the ratio of 1:1 UP1+RGG completely unfolds the Na^+^ form of the G-quadruplex while UP1 was not able to completely unfold the G-quadruplex structure even at 1:1.5 ratio (Figure 8A). Similar observations were made for UP1 titration to the K^+^ form of Tel22 (Supplementary Figure S8).

In case of NMR titrations, the complete unfolding of the Tel22 G-quadruplex was achieved at higher DNA to protein ratio (lower concentration of protein) compared to the CD titrations. The imino protons observed in NMR are from the hydrogen bonds formed between the Hoogsteen base-paired guanine residues in the G-quartets (53). During the unfolding of the G-quadruplex structure, the hydrogen bonds are likely to be disrupted before the disruption of the stacking interaction between the G-quartets. Therefore, this may be the reason that higher protein concentration was required in CD titration to achieve complete unfolding of G-quadruplex structure as compared to NMR titrations, which observe initial disruption of Hoogsteen hydrogen bonded imino protons.

### UP1+RGG efficiently unfolds G-quadruplexes

To gain insight into the relative rate of unfolding of G-quadruplex by UP1+RGG and UP1 we used fluorescence resonance energy transfer (FRET) based kinetic experiment. For this assay, the Tel22 sequence was labeled with two fluorophores, reporter fluorescein (FAM) and quencher tetramethylhodamine (TAMRA) at the 5′ and 3′ ends as the donor and acceptor fluorophores respectively (5′FAM-Tel22-TAMRA3′). In the folded G-quadruplex form of Tel22 these dyes are close in space that results in quenching of FAM fluorescence emission by TAMRA. Upon unfolding of the G-quadruplex structure the distance between the fluorophores is increased resulting in the emission fluorescence of FAM.

Four equivalents of UP1 and UP1+RGG were added individually to a solution of 5′FAM-Tel22-TAMRA3′ prepared in 100 mM KCl or NaCl solution and mixed rapidly. The kinetics of G-quadruplex unfolding by each protein was recorded immediately by measuring the increase of the donor (FAM) fluorescence, Δ*F* (${\rm{\Delta }}F\ = \ {\rm{F}} - {F_0}$, where ${F_0}$ is the FAM fluorescence at 516 nm at *t* = 0 and$\ F$ is the fluorescence at time *t*) at 516 nm (Figure 8B). The unfolding of the quadruplex by the proteins was fitted to single exponential function using Origin 9.0. This revealed that the unfolding of the G-quadruplex by the UP1+RGG (in 100 mM KCl) proceeds with an unfolding rate *k*_obs_= (2.39 ± 0.01) × 10^−4^ s^−1^ as compared to the unfolding by UP1 which proceeds with an unfolding rate of *k*_obs_= (0.45 ± 0.09) × 10^−4^ s^−1^. This clearly shows that UP1+RGG unfolds the K^+^ form of G-quadruplex by ∼5.3 times faster rate compared to UP1. Since the K^+^ form of Tel22 G-quadruplex exists in two mixed conformations (hybrid-1 and hybrid-2), the fluorescence kinetics observed is likely an average of unfolding events of both the conformations. However, even for the Na^+^ form of Tel22 where it adopts a single conformation, we observed that UP1+RGG unfolds the G-quadruplex with a faster rate than UP1 alone (Supplementary Figure S9). This shows that UP+RGG specifically enhances unfolding of DNA G-quadruplex of different conformations and topologies.

Taken together, NMR, CD, and fluorescence results showed that UP1+RGG unfolds DNA G-quadruplex more efficiently compared to UP1 alone. We propose a model in which the RGG-box in UP1+RGG recognizes the structured loops of Tel22 G-quadruplex (Figure 3B) and likely initiates the initial destabilization of the Tel22 G-quadruplex. Simultaneously, it assists in the binding of UP1 to the G-quadruplex to bring about the complete unfolding of the intramolecular G-quadruplex by disrupting the hydrogen bonds and stacking interactions between the G-quartets (Figure 8C).

### Alanine mutation of arginine in the RGG-box of UP1+RGG leads to decreased DNA G-quadruplex unfolding

**Figure 9.**
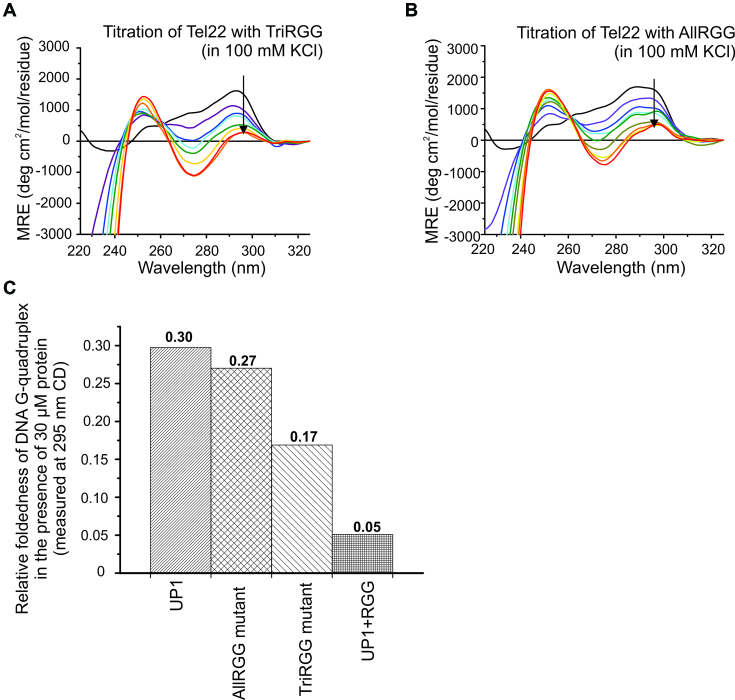
Telomere DNA G-quadruplex unfolding by arginine to alanine mutants of UP1+RGG monitored using CD spectroscopy. (**A, B**) Unfolding of K^+^ form of Tel22 G-quadruplex DNA by TriRGG (A) and AllRGG (B) mutants. The G-quadruplex DNA was titrated with increasing molar excess of proteins. The black arrows in the spectra indicate the gradual decrease in ellipticity at 295 nm with increasing protein concentration. (**C**) The normalized ellipticity at 295 nm of Tel22 at the final titration step (at 1:6 molar ratio of DNA to protein) for UP1, AllRGG, TriRGG and UP1+RGG showing the relative foldedness of the G-quadruplex structure.

According to a classification, RGG-repeats 2, 3 and 4 in hnRNPA1 would constitute a canonical ‘TriRGG’ motif where the RGG-repeats are separated by four residues (54). There is, however, another RGG-repeat (RGG-repeat 1) located nine residues N-terminal to the RGG-repeat 2 (Figure 2A). We mutated ariginine residues to alanine in the tri RGG-motif consisting of repeats 2, 3 and 4 (called the TriRGG-mutant) and in all the four repeats, i.e. RGG-repeats 1, 2, 3 and 4 (called the AllRGG-mutant) in UP1+RGG and expressed and purified the mutant proteins. We used these mutant versions of UP1+RGG proteins and carried out the G-quadruplex unfolding assay using CD spectroscopy (Figure 9A and B). Our results showed that at 1:6 DNA to protein molar ratio, UP1 unfolds G-quadruplex by about 5.8 times less compared to UP1+RGG. The TriRGG-mutant unfolds G-quadruplex by about 3.3-fold less and AllRGG-mutant unfolds by 5.3-fold less compared to UP1+RGG (Figure 9C). These results show that the arginine residues in the four RGG-motifs are important for providing the synergistic effect on DNA G-quadruplex unfolding ability of UP1. This also corroborates our NMR CSP results on RGG-box and Tel22 G-quadruplex interaction that show that three arginine residues in RGG-motifs interact with the G-quadruplex DNA.

## DISCUSSION

Several DNA and RNA binding proteins contain repeats rich in arginine and glycine residues termed as RGG/RG motifs (54–57). More than 1000 human proteins have been found to harbor the RGG/RG motifs, and these proteins influence various physiological processes such as transcription, pre-mRNA splicing, DNA damage signaling, mRNA translation, and the regulation of apoptosis etc. (54–56). Interestingly, other positively charged amino acids lysine and histidine are not found within RGG/RG motifs suggesting that the arginine residues confer distinct properties to the motif besides its positive charge.

Though simple in nature, the RGG-motifs in the context of different proteins have markedly different activities. While primarily the RGG motifs are shown to be the nucleic acid binding motifs, they have also been implicated in mediating the protein-protein interactions (56,58). These protein-protein and protein-nucleic acid interactions are further postulated to be regulated by post-translational modifications especially the methylation of the arginine residues in the RGG-motifs by protein arginine methyltransferases (PRMTs). Isolated RGG-motifs in different proteins have been shown to interact with the single stranded; double stranded, or higher order G-quadruplex forms of both DNA and RNA sequences. These interactions range from being very specific to non-specific in nature and of varying affinities. For example, the RGG-domain in Ewing's sarcoma protein (EWS) was shown to interact with both DNA and RNA G-quadruplexes and the arginine residues in the RGG-domain were shown to be required for this interaction (59). In case of translocated in liposarcoma (TLS) protein, also known as fused in sarcoma (FUS) protein (TLS/FUS) the mutation of tyrosine residues adjacent to RGG-domain to phenylalanine completely shifted the binding specificity of RGG-domain from RNA to DNA G-quadruplex. The tyrosine mutant was shown to further bind DNA with abasic ribose loops but not to abasic deoxyribose loops or to DNA quadruplexes without loops, suggesting that it recognizes the 2′-OH in the ribose sugar in the loops (45). Recently using NMR spectroscopy, further evidences of specific role of pheylalanine (for DNA G-quadruplex) and tyrosine (for RNA G-quadruplex) for G-quadruplex binding were provided (44). In a very recent study, the TLS/FUS RGG3 domain was shown to adopt a β-spiral structure upon binding to G-quadruplexes (60). Structural studies using NMR spectroscopy and X-ray crystallography on the FMRP RGG motif have revealed that the RGG motif gets structured to form a β-turn structure upon binding to an in vitro selected RNA sequence. The RNA sequence formed a structure consisting of a three G-tetrad containing G-quadruplex and an RNA duplex at the stem of the structure. The RGG motif peptide adopts type I β-turn and was shown to bind at the duplex-quadruplex junction (61,62).

Interestingly, the RGG-motifs are often found adjacent to the globular nucleic acid binding domains such as RBDs, RRMs, KH, and ZnF domains etc. For example, the RGG-box in hnRNPA1, FUS, and nucleolin are adjacent to the RRM/RBD domains. Therefore, it is thought that the RGG-motifs can influence the nucleic acid binding functions and other associated activities of the adjacent globular domain (57). The flanking disordered RGG/RG domains were shown to synergistically impart the RNA binding activity to RRM in FUS (63). In case of nucleolin, the C-terminal RGG-motif was shown to cooperate with the adjacent RNA binding domains (RBDs) in stabilizing the c-MYC promoter G-quadruplex. RBD3 and RBD4 together with the RGG-domain were shown to be required for repression of c-MYC transcription (64).

There are previous reports that indicate that the RGG-box is responsible for the cooperative binding of hnRNPA1 to its target DNA or RNA sequences (33–35,65). The ability of UP1 to unfold the DNA G-quadruplex structures is one of its major functions; however the contribution of the RGG-box on the G-quadruplex unfolding activity of UP1 is not well studied. For the first time we provide evidences that in hnRNPA1, the RGG-box influences the DNA G-quadruplex unfolding ability of the UP1 domain. RGG-box does this by binding specifically with the structured G-quadruplex DNA, while it does not interact with the single-stranded DNA. Using NMR chemical shift perturbation experiments, we showed that the binding of the RGG-box is specific to the structured loops in the G-quadruplex DNA. Our NMR CSP results showed that three arginine residues (including assigned R232), four glycine residues, Y244 residue, and a phenylalanine residue along with other residues of the RGG-box are involved in the recognition of G-quadruplex DNA, thereby directly implicating RGG-motif in G-quadruplex recognition. Finally, we propose a model where the RGG-box helps in the initial specific recognition and binding of the G-quadruplex DNA by interacting with the loop nucleotides of the G-quadruplex structure. This brings the UP1 domain close to the G-quadruplex and allows its efficient binding followed by enhanced destabilization of the G-quadruplex structure. Likely two molecules of hnRNPA1 recognize and unfold one G-quadruplex structure (as stoichiometry ratio ‘*n*’ of 2 was observed in ITC titrations). Finally UP1 stabilizes the unfolded form of the DNA by forming high affinity complex with the single-stranded DNA (Figure 8C).

In summary, we showed that the RGG-box imparts the specificity to hnRNPA1 towards G-quadruplex DNA recognition and enhances destabilization of G-quadruplex structure in vitro. In vivo, the effect of RGG-box on hnRNPA1 functions will likely be more pronounced and regulated by post-translational modifications such as methylation, phosphorylation of RGG domain and other proteins.

## Supplementary Material

Supplementary Data
