# Supplementary material for: RGG-box in hnRNPA1 specifically recognizes the telomere G-quadruplex DNA and enhances the G-quadruplex unfolding ability of UP1 domain
Source: Nucleic Acids Res. 2018 Sep 22;46(19):10246–61. doi: 10.1093/nar/gky854 (PMC6212785; doi:10.1093/nar/gky854)
Supplement: Supplementary Data [file gky854_supplemental_files.pdf]

# SUPPLEMENTARY DATA

# RGG-box in hnRNPA1 specifically recognizes the telomere G-quadruplex DNA and enhances the G-quadruplex unfolding ability of UP1 domain

Meenakshi Ghosh[1] and Mahavir Singh[1,2,*]

[1]Molecular Biophysics Unit, Indian Institute of Science, Bengaluru, 560012

[2]NMR Research Centre, Indian Institute of Science, Bengaluru, 560012

* To whom correspondence should be addressed

Tel: +91 80 2293 2839; Fax: +91 80 2293 2839; Email: singh@iisc.ac.in

## Overview:

**SI Figure 1.** Purification of the proteins used in this study.

**SI Figure 2.** Strip plots showing NMR resonance assignment of the residues present in the RGG-box.

**SI Figure 3.** Identification of the phenylalanine and tyrosine residues from selectively $^{15}N$ unlabeled sample of the RGG-box.

**SI Figure 4.** Structural characterization of DNA sequences by CD spectroscopy.

**SI Figure 5.** Imino region of 1D $^{1}H$ NMR spectra of DNA used in this study.

**SI Figure 6.** Arginine residue R#1 undergoes weak chemical shift perturbation upon binding to Tel22 and Tel12.

**SI Figure 7.** A subset of unassigned residues of the RGG-box that show specific fast exchange chemical shift perturbations upon addition of Tel22DNA.

**SI Figure 8.** Titration of $K^+$ form of Tel22 with UP1 and UP1+RGG and monitored through NMR.

**SI Figure 9.** Unfolding of the 5'-FAM and 3'-TAMRA labeled $Na^+$ form of Tel22 (5'FAM-Tel22-TAMRA3') by UP1 and UP1+RGG monitored by observing the emission of FAM at 516 nM.

**SI Table S1.** Individual fit dissociation constants ($K_d$) values determined for residues of the RGG-box for its interaction with Tel22 G-quadruplex DNA.

## Figure S1

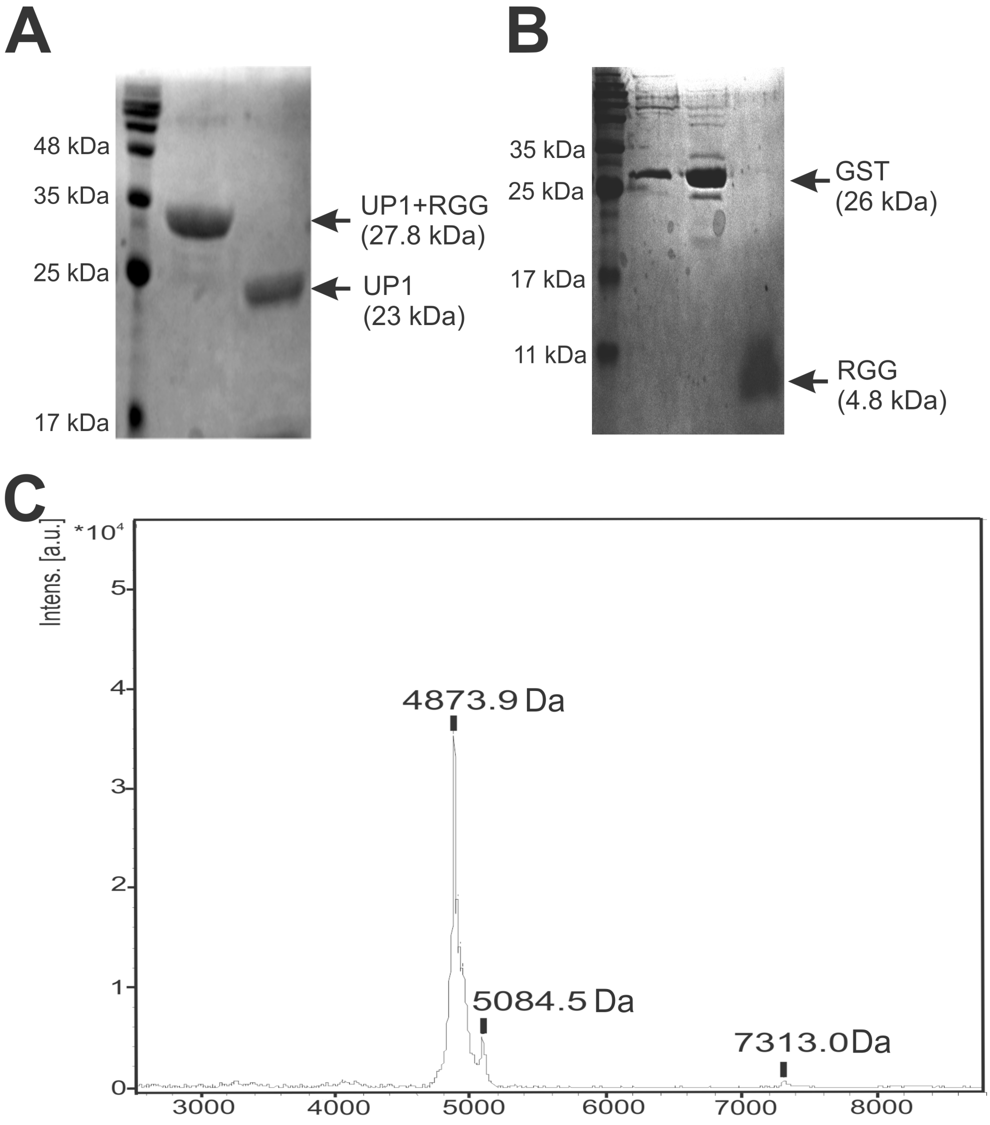


**SI Figure 1.** Purification of the proteins used in this study. **(A)** SDS PAGE showing purified UP1 and UP1+RGG proteins. **(B)** SDS PAGE of purified RGG-box after removal of GST-tag and size exclusion chromatography. **(C)** The mass of the RGG-box was further confirmed by MALDI –TOF MS analysis.

## Figure S2

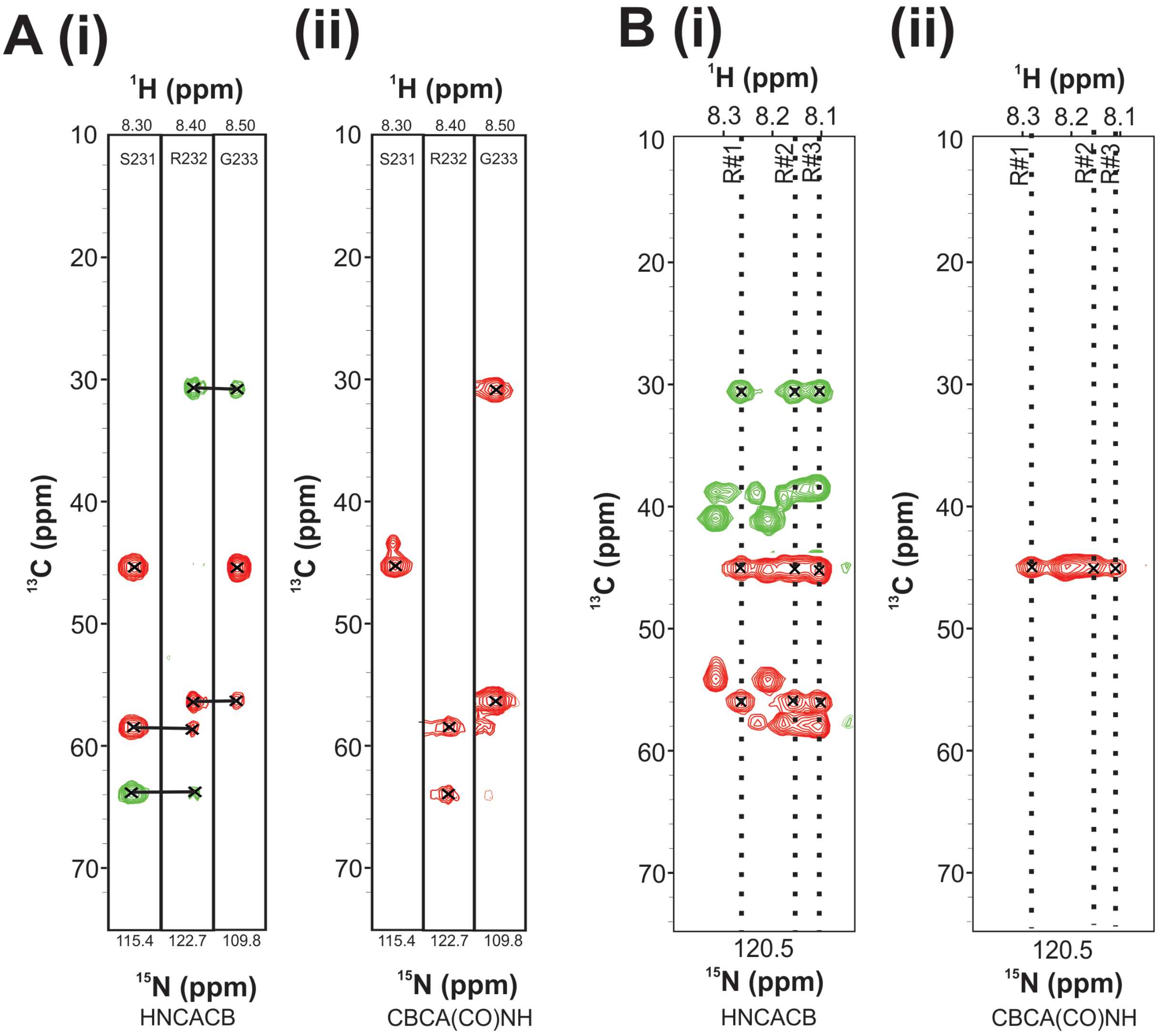


**SI Figure 2.** Strip plots showing NMR resonance assignment of the residues present in the RGG-box. **(A) (i)** HNCACB and **(ii)** CBCA(CO)NH strip plots showing sequential assignment of S231, R232, and G233 residues of the RGG-box. **(B i, ii)** The peaks pertaining to the arginine residues could be identified based on the chemical shift values for the residues and by the presence of the preceding glycine residues in HNCACB/CBCA(CO)NH spectra.

# Figure S3

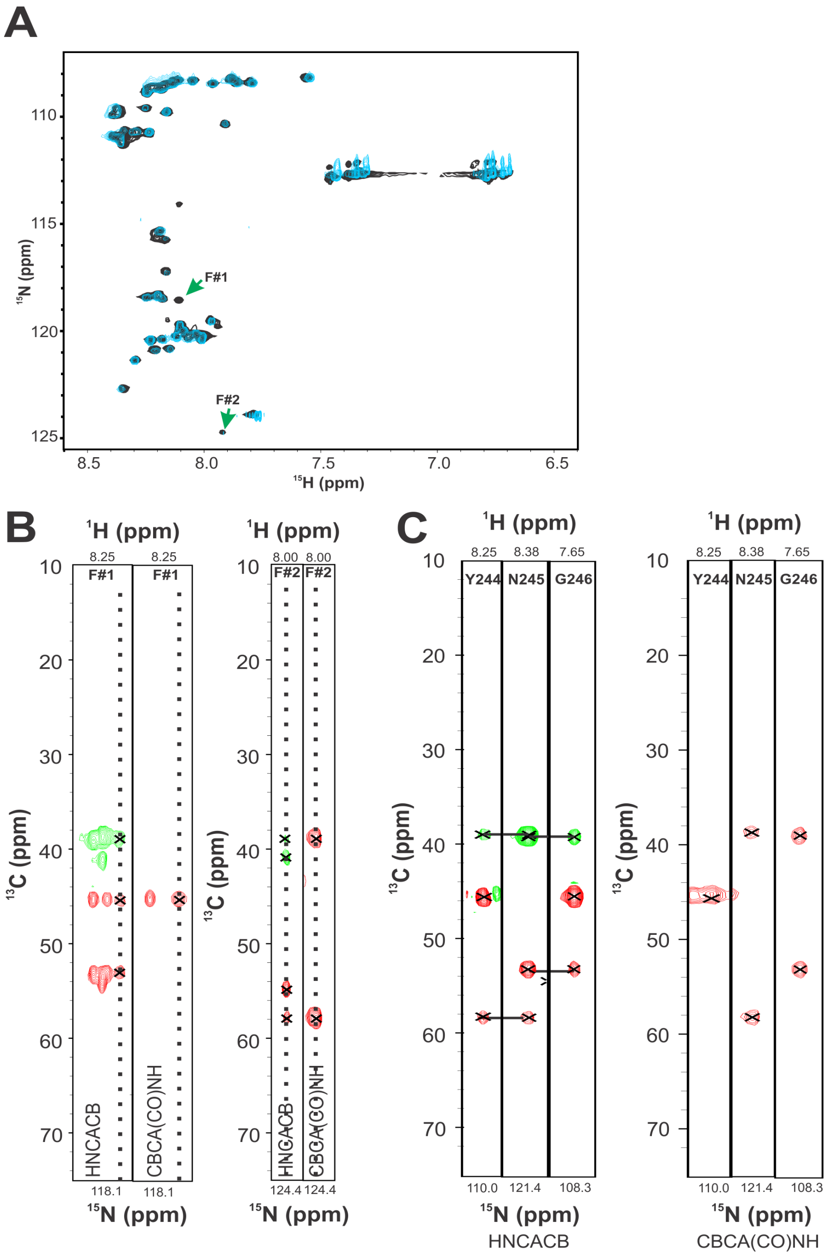


**SI Figure 3.** Identification of the phenylalanine and tyrosine residues from selectively $^{15}$N unlabeled sample of the RGG-box. **(A)** $^{15}$N -$^{1}$H HSQC spectra of selectively Phe unlabeled $^{15}$N RGG-box spectra overlaid on $^{15}$N-$^{1}$H HSQC spectra of $^{15}$N uniformly labeled RGG-box. **(B)** HNCACB and CBCA(CO)NH strip plots of identified Phe residues (F#1 and F#2). **(C)** HNCACB and CBCA(CO)NH strip plots showing the assignment of Y244, N245, G246 residues of the RGG-box.

## Figure S4

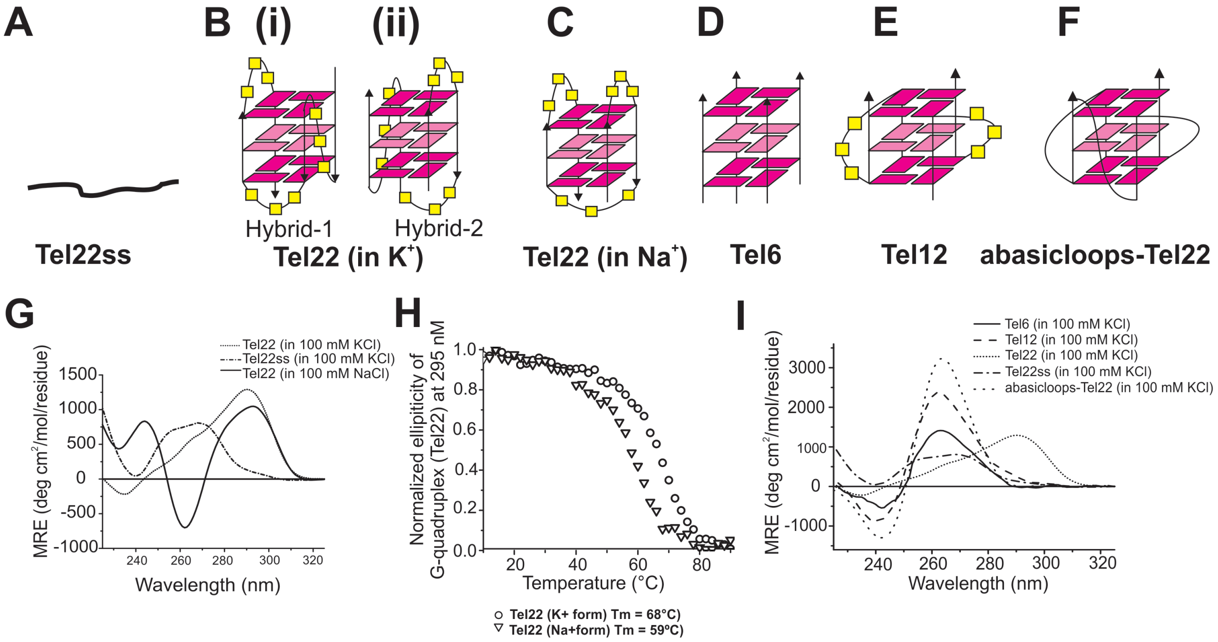


**SI Figure 4.** Structural characterization of DNA sequences by CD spectroscopy. **(A-F)** Cartoon representation of single stranded Tel22ss **(A),** Tel22 intramolecular G-quadruplex in KCl (**B (i) and (ii)**), Tel22 intramolecular G-quadruplex in NaCl (**C**), Tel6 tetrameric G-quadruplex in KCl (**D**), Tel12dimeric G-quadruplex in KCl (**E**), and abasicloops-Tel22 intramolecular G-quadruplex in KCl (**F**). **(G)** CD spectra of Tel22ss and Tel22 in the presence of 100 mM NaCl and KCl. The $Na^+$ form of Tel22 shows positive peaks at 295 nm and 245 nm and a negative peak at 265 nm. The $K^+$ form of Tel22 shows maxima at 295 nm. Tel22ss does not show any of the characteristic peaks of G-quadruplex formation in either NaCl or KCl. **(H)** CD melting curve of $Na^+$ and $K^+$ for of Tel22 G-quadruplex monitored at 295 nm by CD spectroscopy. The melting temperature, $T_m$ deduced for both the forms are mentioned. **(I)** CD spectra of Tel22ss and quadruplexes formed by Tel6, Tel12, Tel22 and abasicloops-Tel22 in 100 mM KCl. Tel22 spectrum is characterized by a positive maxima at 295 nm. $K^+$ form of Tel6, Tel12 and abasicloops-Tel22 spectra are characterized by positive ellipticity at 260 nm and minima at 240 nm. The spectrum of single stranded Tel22ss is also shown.

## Figure S5

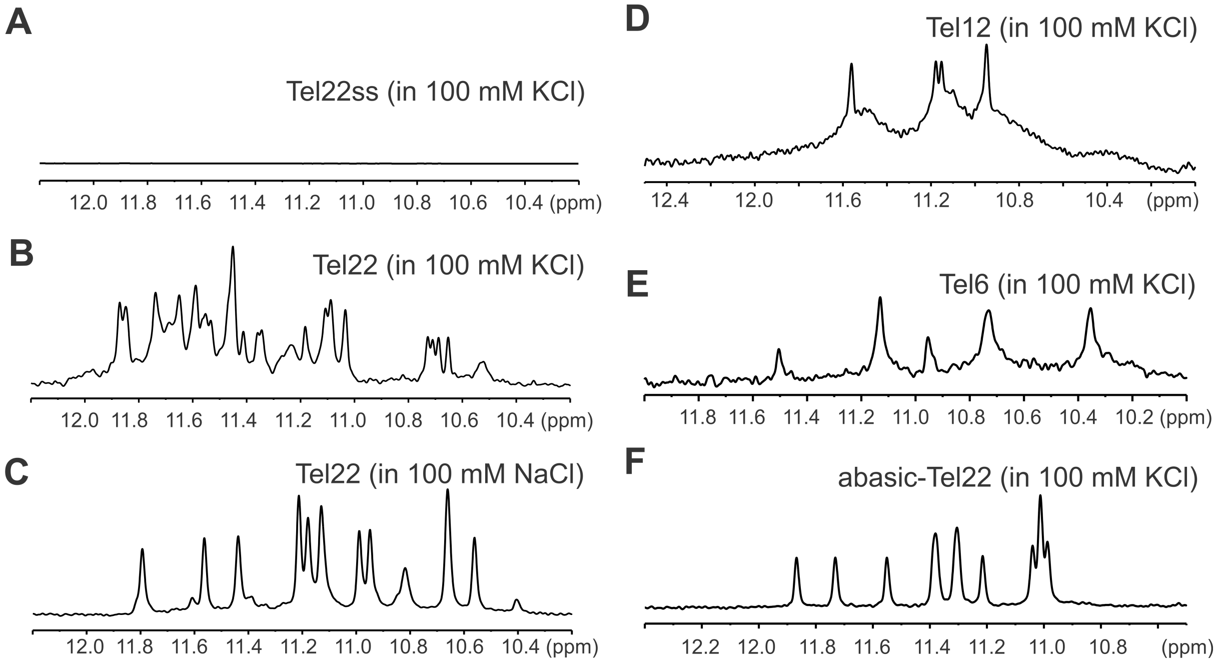


**SI Figure 5.** Imino region of 1D $^{1}$H NMR spectra of DNA used in this study. **(A)** Tel22ss does not show any peak in the imino region, which reinstates the fact that it does not form base-pairs and remains in single stranded conformation. **(B-F)** The imino peaks of G-quadruplex formed byTel22 in 100 mM KCl **(B)**, Tel22 in 100 mM NaCl **(C)**, Tel12 in 100 mM KCl **(D),** Tel6 in 100 mM KCl **(E)** and abasicloops-Tel22 in 100 mM KCl **(F)**.

## Figure S6

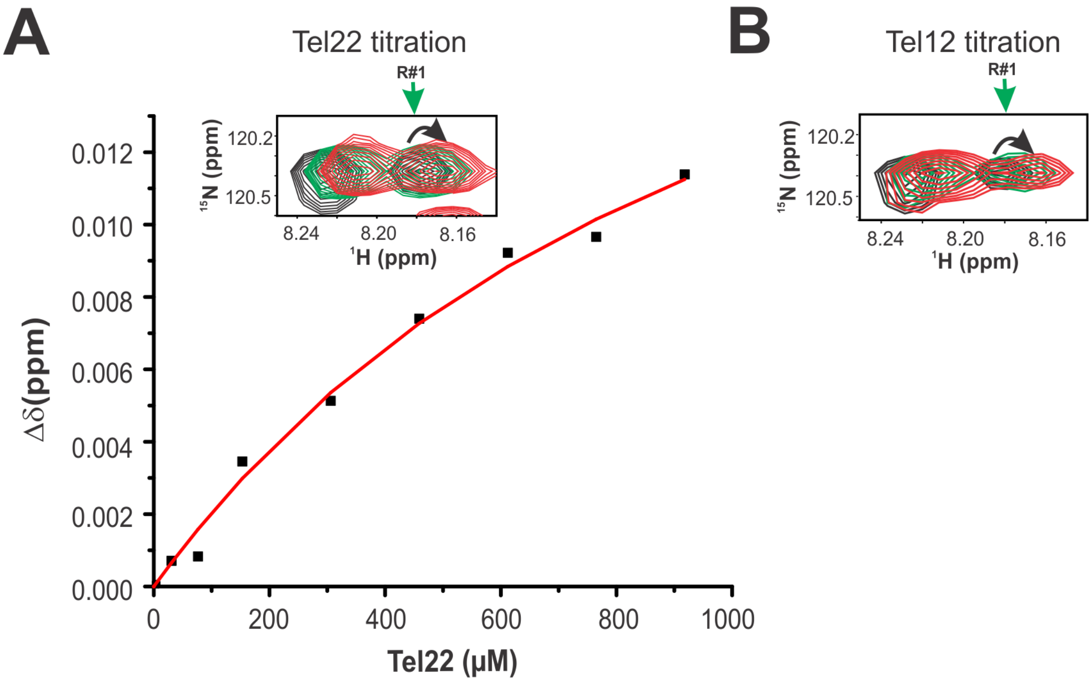


**SI Figure 6.** Arginine residue R#1 undergoes weak chemical shift perturbation upon binding to Tel22 **(A, inset)** and Tel12 **(B)**. The CSPs observed versus added Tel22 DNA concentration for R#1 is plotted. An apparent individual fit $K_d$ of 913.13±33.92 μM was calculated from this fitting.

## Figure S7

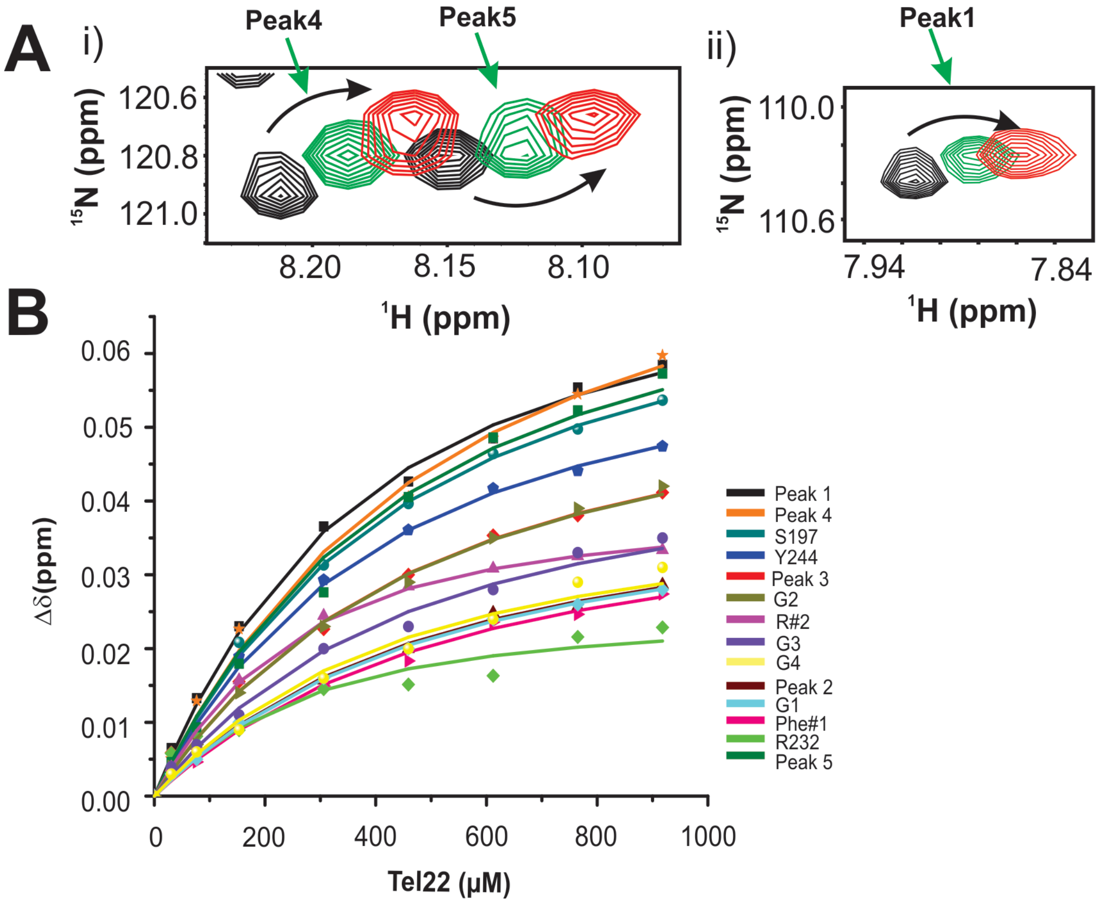


**SI Figure 7.** A subset of unassigned residues of the RGG-box that shows specific fast exchange chemical shift perturbations upon addition of Tel22DNA (**A (i) and (ii)**). **(B)** The CSPs of the residues used for the calculation of the global fit $K_d$ are plotted as a function of added Tel22 DNA.

# Figure S8

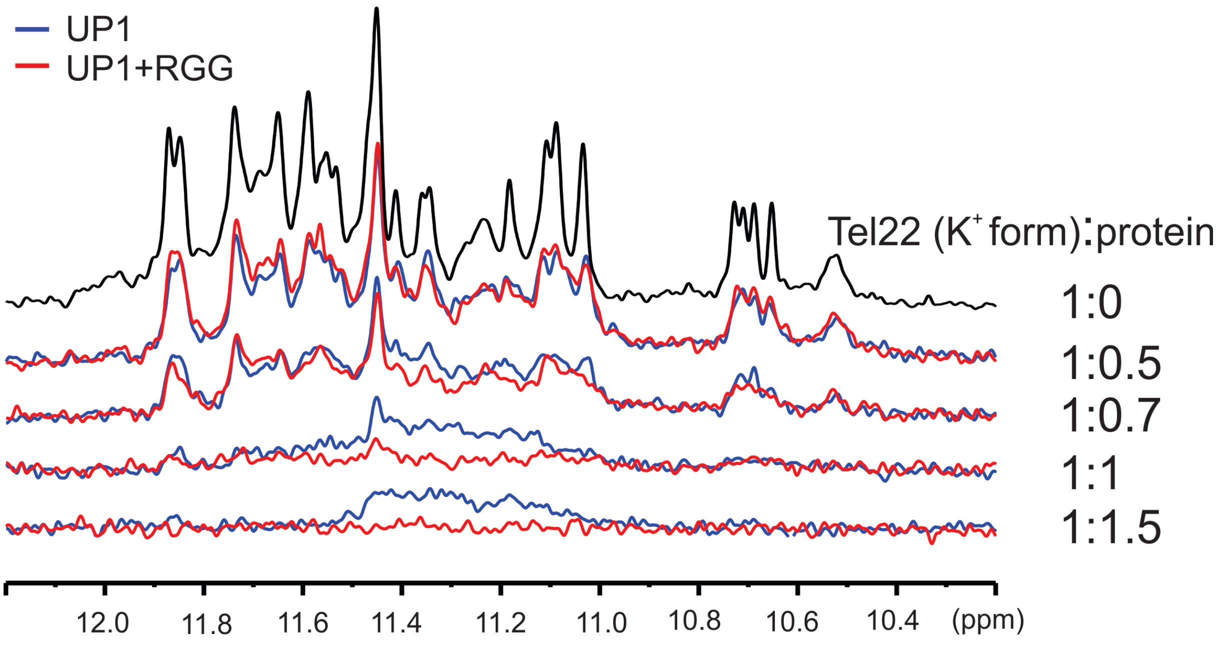


**SI Figure 8.** Titration of $K^+$ form of Tel22 with UP1 and UP1+RGG and monitored through NMR spectroscopy. Imino region of the 1D $^1H$ NMR spectra of $K^+$ form of Tel22 are shown at varying DNA to protein ratios. The DNA was titrated with increasing concentration of UP1 (blue) and UP1+RGG (red).

## Figure S9

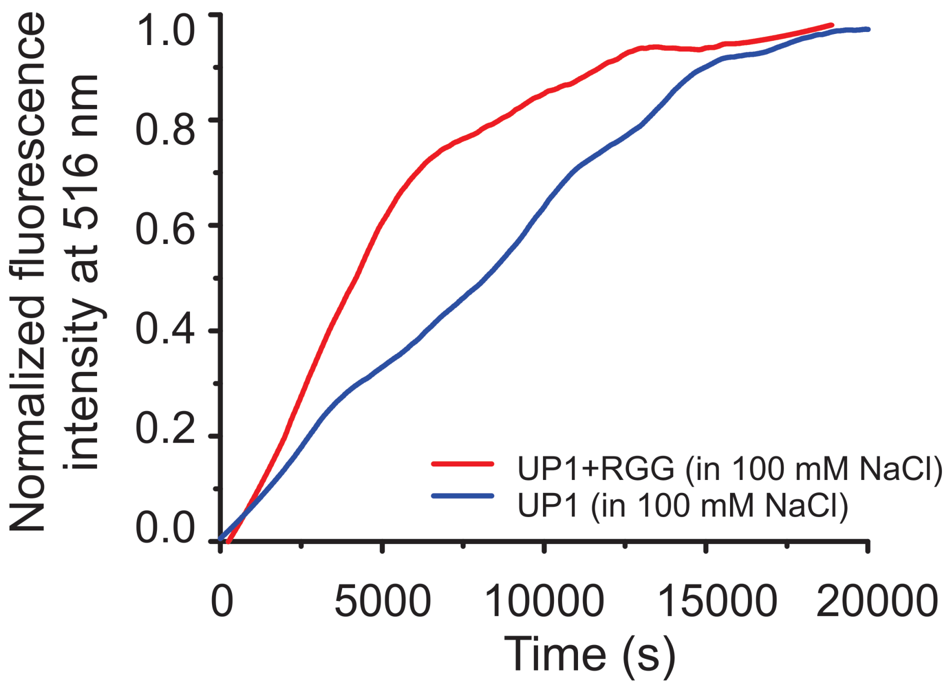


**SI Figure 9.** Unfolding of the 5'-FAM and 3'-TAMRA labeled $Na^+$ form of Tel22 (5'FAM-Tel22-TAMRA3') by UP1 (blue) and UP1+RGG (red) monitored by observing the emission of FAM at 516 nM. 5'FAM-Tel22-TAMRA3' DNA was mixed with 4 molar equivalent of UP1 or UP1+RGG and the emission spectra were recorded over a time period. UP1+RGG proceeds with an unfolding rate of $k_{obs}$= (1.93±0.01) ×$10^{-4}$ $s^{-1}$ and UP1 proceeds with an unfolding rate of $k_{obs}$= (0.63±0.01) ×$10^{-4}$ $s^{-1}$, calculated from a single exponential fit of the data in Origin 9.0.

**Supplementary Table S1.** Individual fit dissociation constant ($K_d$) values determined for residues of the RGG-box for its interaction with Tel22 G-quadruplex DNA based on the observed chemical shift perturbations.

| ***Sl. No.*** | ***Residues*** | ***Individual fit $K_d$ (in µM)*** |
|---|---|---|
| 1 | Peak1 | 263.89±9.19 |
| 2 | Peak2 | 399.43±13.72 |
| 3 | Peak3 | 375.07±15.49 |
| 4 | Peak4 | 406.1±15.9 |
| 5 | Peak5 | 349.5±19.7 |
| 6 | F#1 | 432.86±15.56 |
| 7 | S197 | 346.49±10 |
| 8 | R232 | 171.81±34.36 |
| 9 | Y244 | 305.18±12.926 |
| 10 | R#2 | 150.76±7.31 |
| 11 | G#1 | 404.96±7.81 |
| 12 | G#2 | 377.23±9.89 |
| 13 | G#3 | 348.67±18.08 |
| 14 | G#4 | 342.60±23.44 |
| **Global fit $K_d$ for all these residue: 349±35 µM** | | |
